# Nanocasting SiO_2_ into metal–organic frameworks imparts dual protection to high-loading Fe single-atom electrocatalysts

**DOI:** 10.1038/s41467-020-16715-6

**Published:** 2020-06-05

**Authors:** Long Jiao, Rui Zhang, Gang Wan, Weijie Yang, Xin Wan, Hua Zhou, Jianglan Shui, Shu-Hong Yu, Hai-Long Jiang

**Affiliations:** 1 Hefei National Laboratory for Physical Sciences at the Microscale, CAS Key Laboratory of Soft Matter Chemistry, Collaborative Innovation Center of Suzhou Nano Science and Technology, Department of Chemistry, University of Science and Technology of China, Hefei, Anhui, 230026 People’s Republic of China; 2 Materials Science Division, Argonne National Laboratory, Lemont, IL 60439 USA; 3 School of Energy and Power Engineering, North China Electric Power University, Baoding, 071003 People’s Republic of China; 4 School of Materials Science and Engineering, Beihang University, Beijing, 100083 People’s Republic of China; 5 X-ray Science Division, Advanced Photon Source, Argonne National Laboratory, Lemont, IL 60439 USA

**Keywords:** Catalyst synthesis, Electrocatalysis, Heterogeneous catalysis, Metal-organic frameworks, Electrocatalysis

## Abstract

Single-atom catalysts (SACs) have sparked broad interest recently while the low metal loading poses a big challenge for further applications. Herein, a dual protection strategy has been developed to give high-content SACs by nanocasting SiO_2_ into porphyrinic metal–organic frameworks (MOFs). The pyrolysis of SiO_2_@MOF composite affords single-atom Fe implanted N-doped porous carbon (Fe_SA_–N–C) with high Fe loading (3.46 wt%). The spatial isolation of Fe atoms centered in porphyrin linkers of MOF sets the first protective barrier to inhibit the Fe agglomeration during pyrolysis. The SiO_2_ in MOF provides additional protection by creating thermally stable FeN_4_/SiO_2_ interfaces. Thanks to the high-density Fe_SA_ sites, Fe_SA_–N–C demonstrates excellent oxygen reduction performance in both alkaline and acidic medias. Meanwhile, Fe_SA_–N–C also exhibits encouraging performance in proton exchange membrane fuel cell, demonstrating great potential for practical application. More far-reaching, this work grants a general synthetic methodology toward high-content SACs (such as Fe_SA_, Co_SA_, Ni_SA_).

## Abstract (web summary)

Single-atom catalysts (SACs) with high metal loading are highly desired to improve catalytic performance. Here, the authors report a dual protection strategy by nanocasting SiO_2_ into metal–organic frameworks to prepare high-loading SACs with excellent catalytic performance toward oxygen reduction.

## Introduction

Single-atom catalysts (SACs), with the maximal utilization of metal atoms, open up a new frontier and attract increasing attention in catalysis^1–10^. Integrated with plenty of merits, including highly dispersed sites, high activity, excellent selectivity, and good reusability, SACs have been regarded as an ideal platform to bridge the gap between homogenous and heterogeneous catalysts^1–4^. Nevertheless, isolated metal atoms in SACs tend to agglomerate due to the high surface energy. Though significant progress has been achieved to ensure the atomic dispersion of metal atoms, metal loadings of SACs are basically low (<1 wt%). The construction methodology of stable SACs, especially in high metal loadings, is highly desired yet remains a grand challenge^11–13^. In addition, to boost the catalytic performance of SACs, their physical features, including porous structure and surface area, which dominate the accessibility to active sites, should also be optimized^14–16^.

Metal–organic frameworks (MOFs)^17–25^, featuring well-defined and tailored structures, present particular advantages in the precise fabrication of catalysts, especially SACs^26–33^. The present synthetic strategy for MOF-based SACs is to augment the distance between adjacent metal atoms based on the mixed metal/ligand and pore confinement, which effectively inhibits the agglomeration of metal atoms under pyrolysis^27–30^. Unfortunately, these strategies cause the decrease of metal loadings. As a result, even bearing the structural advantages in MOF-based SACs, their metal loadings are unsatisfying, such as Fe_SA_ (usually, <2 wt%)^26–33^. In addition, most of reported MOF-derived SACs possess micropores (<2 nm), which is unfavorable to mass transfer in catalytic process^27,30^. To address these issues, alternative synthetic strategies for MOF-derived SACs are imperative to improving the metal loadings and pore structures.

To realize SACs with high metal loadings, the main obstacle to overcome is their easy-to-migrate feature due to their high surface energy, especially under pyrolysis. It was found that inorganic silica (SiO_2_) protection method can stabilize metal nanoparticles/clusters by decreasing surface energy of metal atoms^1,34–38^, which might be effective toward the stabilization of highly loaded SACs. Making full use of the porosity of MOFs, SiO_2_ can be easily nanocasted into the pore space of MOFs to interact with the isolated metal atoms on MOF skeleton, which would significantly lower their surface energy_._ In consequence, the introduction of SiO_2_ into MOFs, when integrated with the merits of MOFs, should be a very promising route to improve metal loadings in SACs.

**Fig. 1:**
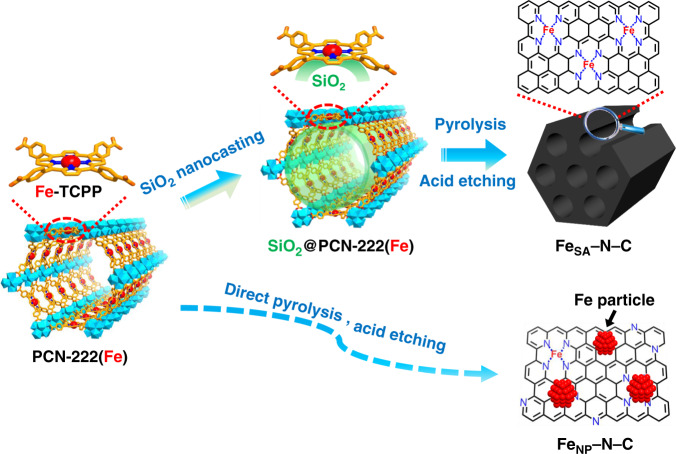
Schematic illustration. Illustration showing the nanocasting-assisted fabrication of Fe_SA_–N–C from PCN-222(Fe).

In this work, we creatively put forward a nanocasting strategy to introduce SiO_2_ into the mesopores of a porphyrinic MOF, PCN-222(Fe), featuring single Fe(III) site in each porphyrin linker^39–41^. Thanks to the 1D mesochannel with a diameter of ~3.2 nm, SiO_2_ can be sufficiently filled into PCN-222(Fe), forming thermal stable FeN_4_/SiO_2_ interfaces. Upon high-temperature pyrolysis and SiO_2_ removal, the single-atom Fe catalyst, denoted Fe_SA_–N–C, with a Fe loading as high as 3.46 wt%, is obtained (Fig. 1). During the pyrolysis, the spatial isolation of Fe atoms anchored by N atoms in porphyrin linkers is the first protective barrier to inhibit the Fe agglomeration. The silica in MOF channels serves as oxide substrate to interact with Fe atoms that can increase migration energy barrier of Fe atoms and prevent their aggregation. Meanwhile, upon removal of silica, the porosity and surface area of the resultant N-doped porous carbon can be improved, benefiting the exposure of active sites and mass transfer. As a result, the optimized Fe_SA_–N–C exhibits excellent oxygen reduction reaction (ORR) performance, surpassing the state-of-the-art Pt/C, and almost all reported non-noble-metal catalysts, in both alkaline and the more challenging acidic solutions. Significantly, the Fe_SA_–N–C reaches a current density of 292 mA cm^−2^ at 0.8 V and the highest power density of 0.68 W cm^−2^, comparable to that of the best non-noble metal catalysts, in H_2_–O_2_ proton exchange membrane fuel cell (PEMFC).

## Results

### Synthesis and characterization of Fe_SA_–N–C

**Fig. 2:**
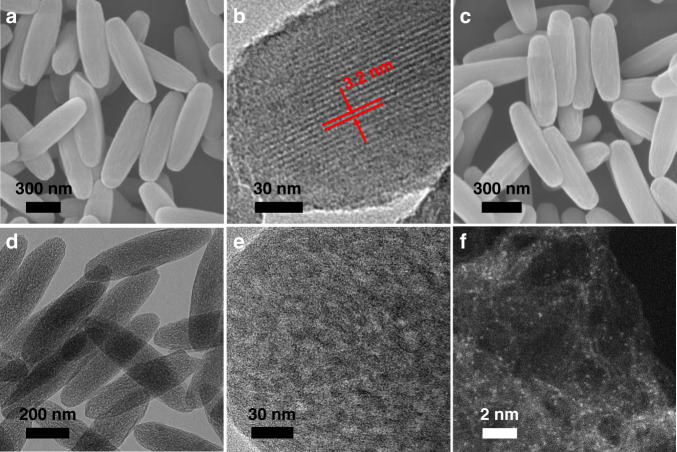
Microscopic characterizations. **a** Scanning electron microscopy (SEM) and **b** transmission electron microscopy (TEM) images of PCN-222(Fe). **c** SEM and **d** TEM images of Fe_SA_–N–C. **e** Enlarged TEM image showing the mesoporous structure of Fe_SA_–N–C. **f** Aberration-corrected HAADF-STEM image of Fe_SA_–N–C.

The PCN-222(Fe) was prepared by employing trifluoroacetic acid (TFA), instead of the traditionally used benzoic acid, as a modulator (Supplementary Fig. 1)^39^. The scanning electron microscopy (SEM) image of PCN-222(Fe) presents the uniform spindle morphology with a diameter ~250 nm (Fig. 2a). The low boiling point of TFA makes it easy to be removed by direct degassing without additional pre-activation process (necessary for benzoic acid modulator) to deliver available pore space in PCN-222(Fe) (Supplementary Table 1). The N_2_ sorption with a typical type-IV isotherms present a high surface area up to 2040 m^2^ g^−1^ and the pore size distribution suggests the mesochannels centered at 3.2 nm, in good agreement with the transmission electron microscopy (TEM) observation (Fig. 2b, Supplementary Fig. 2). This greatly facilitates the subsequent introduction of tetraethylorthosilicate (TEOS) into PCN-222(Fe) mesopores for SiO_2_ nanocasting, after degassing the MOF at 150 °C. The facile TFA removal and mesoporosity guarantee the sufficient percolation of TEOS through the entire inner space of PCN-222(Fe). Upon HCl vapor treatment, TEOS in PCN-222(Fe) can be hydrolyzed and condensed to silica, affording SiO_2_@PCN-222(Fe) composite with well-retained MOF crystallinity, thanks to the ultrahigh acidic stability of the MOF (Supplementary Fig. 1)^39^.

The infrared peak at 1090 cm^−1^ assignable to Si-O-Si clearly confirms the SiO_2_ formation (Supplementary Fig. 3)^37^. The reduced surface area (1190 m^2^ g^–^^1^) and mesopore size (2.9 nm) in the composite, in reference to the parent MOF (BET of 2040 m^2^ g^−1^; pore size of 3.2 nm), further indicate the successful infiltration of SiO_2_ in PCN-222(Fe). The elemental mapping images of SiO_2_@PCN-222(Fe) further illustrate the homogeneous dispersion of Si in PCN-222(Fe) (Supplementary Fig. 4). After the pyrolysis of SiO_2_@PCN-222(Fe) at 800 °C, the composite of metal (oxides) stabilized by porous carbon is produced. Upon the removal of the oxide by acid etching, Fe_SA_–N–C with retained spindle morphology is finally obtained and no particles are observed in TEM images (Fig. 2c–e, Supplementary Fig. 5a). The elemental mapping images clearly demonstrate the homogenous dispersion of Fe and N elements on Fe_SA_–N–C (Supplementary Fig. 6). Aberration-corrected high-angle annular darkfield scanning transmission electron microscope (HAADF-STEM) observation shows the isolated and high-density bright spots, implying the formation of single metal atoms (Fig. 2f, Supplementary Fig. 5b). The accurate contents of Fe (3.46 wt%) and N (4.87 wt%) have been quantified by inductively coupled plasma atomic emission spectrometry (ICP) and elemental analysis, demonstrating the presence of single Fe atom and suggesting the higher Fe loading than almost all reported single-atom Fe-incorporated carbon-based materials (Supplementary Table 2). Moreover, the quantitative analysis of X-ray photoelectron spectroscopy (XPS) and energy-dispersive spectroscopy further confirm the high loading of Fe, in accordance with the ICP results (Supplementary Table 3). In addition, the content of Zr is extremely low, illustrating Fe is the dominated metal species in Fe_SA_–N–C (Supplementary Table 3). As illustrated above, the perfect combination of PCN-222(Fe) and SiO_2_ can exert their respective advantages for the creation of the resultant Fe_SA_–N–C, which are visually summarized (Supplementary Fig. 7).

Powder X-ray diffraction (PXRD) pattern of Fe_SA_–N–C gives two broad peaks in the ranges of 20–30° and 40–45°, corresponding to (002) and (101) planes of graphitized carbon, and no diffraction of Fe-based species is identifiable, in accordance with the TEM observation results (Fig. 2c–e, Supplementary Fig. 8). Raman scattering spectrum for Fe_SA_–N–C gives low intensity ratio (*I*_D_/*I*_G_ = 0.95) of D band (~1345 cm^−1^) and G band (~1590 cm^−1^), illustrating the high graphitization degree (Supplementary Fig. 9). N_2_ sorption measurement for Fe_SA_–N–C manifests its high BET surface area up to 1615 m^2^ g^−1^ (Supplementary Fig. 10). The mesoporous pore size distribution extends up to 10 nm (Supplementary Fig. 10), which can be also seen in enlarged TEM image (Fig. 2e). The large surface area and hierarchical pore of Fe_SA_–N–C would make the single Fe atoms readily accessible and guarantee high-flux mass transfer, which are essential to boost the catalysis^42^. The existing states of Fe and N elements have been examined by XPS. The N 1 s XPS spectrum of Fe_SA_–N–C is fitted into five configurations, including pyridinic N (398.5 eV), Fe–N (399.2 eV), pyrrolic N (400.2 eV), graphitic N (401.1 eV), and oxidized N (402.9 eV), respectively (Supplementary Fig. 11a)^27–29,34–38,43^. The Fe 2 p_3/2_ binding energy in Fe_SA_–N–C centers at 710.5 eV (close to Fe^3+^), suggesting the positively charged Fe atoms (Supplementary Fig. 11b). No Fe^0^ belonging to Fe particle can be identified from XPS spectrum, in consistent with the absence of Fe particles from PXRD and TEM results (Fig. 2d–f, Supplementary Fig. 8). In addition, no obvious Si residual can be detected from the XPS result of Fe_SA_–N–C (Supplementary Fig. 12). It is noteworthy that the direct pyrolysis of PCN-222(Fe) without SiO_2_ leads to the formation of Fe nanoparticles in the resultant catalyst (denoted Fe_NP_–N–C), manifesting the important role of SiO_2_ in inhibiting the agglomeration of Fe atoms under pyrolysis (Supplementary Fig. 13). The absence of Fe peaks in the XRD pattern of Fe_NP_–N–C should be due to the small amount of Fe NPs (Supplementary Fig. 8).

### X-ray absorption spectroscopy studies

**Fig. 3:**
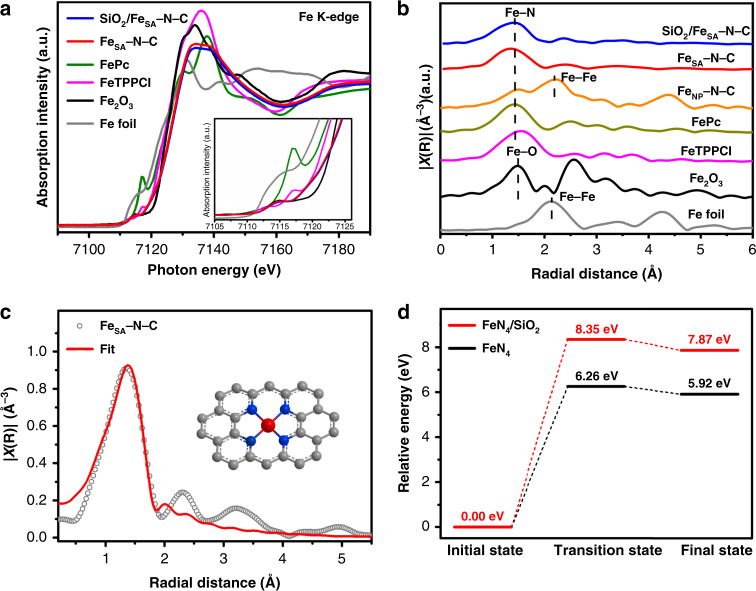
Structure characterizations and DFT calculations. **a** Fe K-edge XANES and **b** FT-EXAFS spectra of SiO_2_/Fe_SA_–N–C, Fe_SA_–N–C, and Fe_NP_–N–C (represented by blue, red, and orange lines, respectively). **c** EXAFS fitting for Fe_SA_–N–C (red line: fitting curve; gray cycles: experimental data). Inset: schematic model of Fe coordination environment in Fe_SA_–N–C. The red, blue, and gray spheres represent Fe, N, and C atoms, respectively. **d** The relative energy along the intrinsic reaction coordinate for FeN_4_/SiO_2_ (red line) and FeN_4_ (black line) migrating from initial state to final state.

To gain more information of the electronic structure and coordination environment of single Fe atoms in Fe_SA_–N–C, X-ray absorption near-edge structure (XANES) and Fourier transform-extended X-ray absorption fine structure (FT-EXAFS) spectra have been examined. The XANES spectra of Fe in SiO_2_/Fe_SA_–N–C and Fe_SA_–N–C show almost the same absorption edge located at between Fe foil and Fe_2_O_3_ (Fig. 3a), illustrating the positive valence state of Fe close to +3. The Fourier transformed EXAFS spectra of both SiO_2_/Fe_SA_–N–C and Fe_SA_–N–C present a dominated peak at ~1.4 Å respecting to the Fe–N scattering path, and no Fe–Fe bond is detected (Fig. 3b). Furthermore, the curve fitting for EXAFS data of Fe_SA_–N–C further verifies the coordination structure around Fe centers. The best fitting result for the first shell indicates that Fe atoms are fourfold coordinated by N atoms in average (Fig. 3c, Supplementary Fig. 14, Supplementary Table 4).

### Insight into the formation process of Fe_SA_–N–C

To unveil the critical role of the SiO_2_ nanocasting for the generation of atomically dispersed Fe in Fe_SA_–N–C, as a control, pure PCN-222(Fe) without SiO_2_ was directly pyrolyzed at 800 °C in N_2_ atmosphere. The Fe-based particles with clear Fe–Fe bond are observed in the EXAFS spectrum of Fe_NP_–N–C, in stark contrast to that of Fe_SA_–N–C derived from SiO_2_@PCN-222(Fe) (Fig. 3b). The results above unambiguously demonstrate the crucial role of SiO_2_ in the prevention of Fe atom migration/growth at a high loading under pyrolysis. To further illustrate the stabilizing effect of SiO_2_ for Fe atoms in SiO_2_/Fe_SA_–N–C, the energy changes along the intrinsic reaction coordinate were also investigated through density functional theory (DFT) calculation. By selecting the FeN_4_ and FeN_4_/SiO_2_ as representative models for Fe_SA_–N–C and SiO_2_/Fe_SA_–N–C, respectively, the migration energies of each model from the initial state to the final state have been studied in detail (Supplementary Figs. 15–17). The migration energy barrier of Fe atom in FeN_4_/SiO_2_ is found to be 8.35 eV, obviously larger than 6.26 eV of FeN_4_ without SiO_2_, suggesting the more thermally stable Fe atoms in SiO_2_/Fe_SA_–N–C due to the stabilization effect of SiO_2_ for Fe atoms (Fig. 3d). Moreover, SiO_2_ also serves as a hard template, creating much larger surface area of Fe_SA_–N–C (1615 m^2^ g^−1^) than those of Fe_NP_–N–C (445 m^2^ g^−1^) and the N-doped porous carbon (simply as N–C, 612 m^2^ g^−1^) derived by the pyrolysis of PCN-222 without Fe centers (Supplementary Fig. 10). Therefore, the SiO_2_-assisted MOF pyrolysis strategy is able to control the dispersion of active sites and tailor the microstructure of Fe_SA_–N–C, which would be of great significance for subsequent catalysis.

Based on the results above, the formation process of Fe_SA_–N–C from SiO_2_@PCN-222(Fe) is readily understood. The periodic array of porphyrin linkers in the MOF guarantees the Fe isolation. The N atoms in porphyrin linker serve as the first safeguard to stabilize the Fe species. Moreover, the SiO_2_ in the MOF pores behaves as an oxide support to further offer anchoring effect, upraising migration energy barrier of Fe atoms, and preventing their migration/growth upon pyrolysis (Fig. 3d). Based on the synergistic interactions endowed by the MOF and SiO_2_, the thermal agglomeration of Fe atoms is suppressed, leading to the high Fe loadings in atomically dispersed form in Fe_SA_–N–C. In addition to Fe_SA_–N–C, Co_SA_–N–C, and Ni_SA_–N–C have also been successfully fabricated from SiO_2_-nanocasted PCN-222(Co) and PCN-222(Ni), respectively, further manifesting the reliability and universality of this powerful strategy (Supplementary Fig. 18).

### Electrocatalytic performance for ORR and fuel cells

**Fig. 4:**
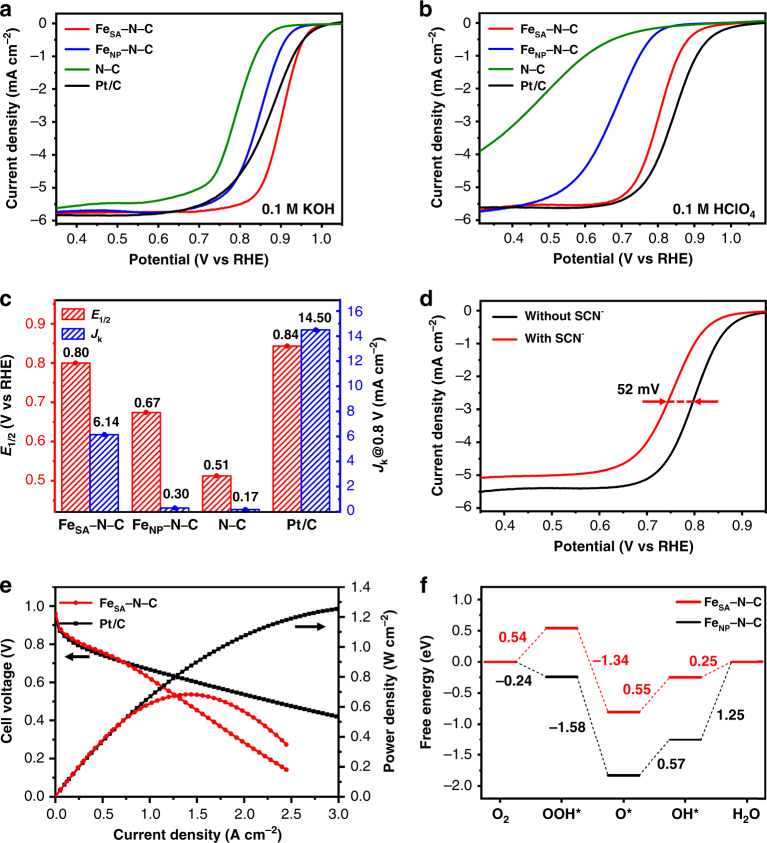
Electrochemical performances and DFT calculations. LSV curves for Fe_SA_–N–C (red line), Fe_NP_–N–C (blue line), N–C (olive line) and Pt/C (black line) in **a** 0.1 M KOH and **b** 0.1 M HClO_4_. **c** Comparison of *E*_1/2_ and *J*_k_ at 0.80 V for various catalysts in 0.1 M HClO_4_. **d** LSV curves of Fe_SA_–N–C in 0.1 M HClO_4_ before (black line) and after (red line) the addition of SCN^−^. **e** Polarization and power density curves of PEMFCs with Fe_SA_–N–C (red dots) and Pt/C (black dots) cathode catalysts. **f** Free energy diagrams of ORR on Fe_SA_–N–C (red line) and Fe_NP_–N–C (black line) in acidic media (pH = 1).

Encouraged by the high-content single-atom Fe sites and pore structure of Fe_SA_–N–C, its ORR performance has been investigated. To our delight, when firstly tested in 0.1 M KOH solution, Fe_SA_–N–C exhibits the highest half-wave potential (*E*_1/2_ = 0.90 V) and kinetic current density (*J*_k_) at 0.85 V (37.19 mA cm^−2^) among Fe_NP_–N–C, N–C and the commercial Pt/C. The mass activity of Fe_SA_–N–C is calculated to be 21.36 mA g^−1^ at 0.9 V, much better than Fe_NP_–N–C and N–C counterparts (Supplementary Table 5). The ideal 4e^−^ transfer process, as well as the excellent durability and methanol tolerance of Fe_SA_–N–C all manifest the superior performance to Pt/C and most non-noble metal catalysts ever reported under alkaline conditions (Fig. 4a, Supplementary Figs. 19–22, Supplementary Table 6). Encouraged by the excellent ORR performance of Fe_SA_–N–C in alkaline media, we further explore its performance under more challenging acidic conditions. When tested in 0.1 M HClO_4_, the linear sweep voltammetry (LSV) curve of Fe_SA_–N–C shows remarkable ORR activity with much higher *E*_1/2_ (0.80 V) than that of Fe_NP_–N–C (0.67 V), and N–C (0.51 V; Fig. 4b, c). Also, Fe_SA_–N–C shows better mass activity 1.12 mA g^−1^ at 0.9 V than that of Fe_NP_–N–C and N–C counterparts (Supplementary Table 7). In addition, Fe_SA_–N–C demonstrates superior *J*_k_ at 0.80 V (6.14 mA cm^−2^) to that of Fe_NP_–N–C (0.30 mA cm^−2^) and N–C (0.17 mA cm^−2^), revealing the more favorable kinetics of Fe_SA_–N–C (Fig. 4c). The superior performance of Fe_SA_–N–C to Fe_NP_–N–C and N–C clearly manifests that single-atom Fe sites are the real origin of the high activity for ORR. It is worth noting that, although much endeavor has been devoted, very limited non-noble metal catalysts were reported to present excellent ORR performance in acidic media. The results highlight the particular superiority of Fe_SA_–N–C toward ORR among all reported non-noble-metal catalysts (Supplementary Fig. 23, Supplementary Table 8). Given the superb activity of Fe_SA_–N–C, the deeper ORR investigations have been further conducted in HClO_4_. To understand the electron transfer mechanism, LSV curves at different rotating rates of rotating disk electrode are recorded. The Koutechy-Levich (K-L) plots obtained from the LSV curves present good linearity, manifesting the first-order reaction kinetics for Fe_SA_–N–C with a potential-independent electron transfer rate (Supplementary Fig. 24a)^44,45^. The electron transfer number calculated by K-L equation is determined to be 4, in accordance with the result of the rotating ring disk electrode test (Supplementary Fig. 24a, b). Furthermore, the LSV curves of Fe_SA_–N–C after 20,000 cycles, and the methanol addition present its excellent durability and methanol tolerance, in stark contrast to the significant decline of Pt/C (Supplementary Figs. 25 and 26). The XPS spectrum of Fe for Fe_SA_–N–C after stability test shows identical peak to the as-prepared catalyst (Supplementary Fig. 27). From the aberration-corrected HAADF-STEM image, Fe atoms in Fe_SA_–N–C after stability test still maintain atomic dispersion on the porous carbon (Supplementary Fig. 28). The results above manifest that the single Fe atoms in Fe_SA_–N–C can retain the structure after stability test. In addition, to ascertain the critical role of single Fe atoms for the excellent ORR, SCN^−^, with strong affinity to Fe, was employed to act as a probe to poison Fe–N sites^46,47^. Upon the addition of KSCN solution into 0.1 M HClO_4_, the deactivation of Fe_SA_–N–C for ORR, with the half-wave potential decreased significantly by 52 mV, clearly manifests that single Fe atoms are responsible for the excellent ORR performance of Fe_SA_–N–C (Fig. 4d). The excellent ORR activity in acid media of Fe_SA_–N–C has been further approved by the PEMFC measurements. The Fe_SA_–N–C produces a remarkable current density of 292 mA cm^−2^ at 0.8 V (or 463 mA cm^−2^ at 0.8 V_iR-free_), which is among the highest activities of platinum group metals-free cathodes reported in real PEMFCs (Fig. 4e, Supplementary Fig. 29, Supplementary Table 9). Moreover, the peak power density reaches a considerable value of 0.68 W cm^−2^, ~54% the power density of the Pt-cathode under the same operating conditions (Fig. 4e). The fuel cell stability test for Fe_SA_–N–C indicates a stabilized current density ~0.3 A cm^−2^ after 20 h testing at 0.5 V (Supplementary Fig. 30). Furthermore, SiO_2_@Fe_SA_–N–C, featuring much smaller pore size and volume than Fe_SA_–N–C, has also been tested for fuel cell application (Supplementary Fig. 31). It can be seen that SiO_2_@Fe_SA_–N–C shows much inferior performance to Fe_SA_–N–C, clearly demonstrating the vital importance of porous structure in Fe_SA_–N–C for the improvement of fuel cell performance (Supplementary Fig. 32).

To better understand the extraordinary ORR activity of Fe_SA_–N–C, DFT calculations have been performed to obtain the free energy diagrams of Fe_SA_–N–C at equilibrium potential based on the four elementary steps of ORR in acidic media (Fig. 4f, Supplementary Figs. 33–35, Supplementary Tables 10–12). It is clear that Fe_SA_–N–C and Fe_NP_–N–C show different ORR rate-determining steps. For Fe_SA_–N–C, the formation of OH* is the most sluggish step with the highest uphill free energy change of 0.55 eV. As for Fe_NP_–N–C, the rate-determining step is the last electron transfer step, requiring the energy change of 1.25 eV. Obviously, the resistance of ORR on Fe_SA_–N–C is much smaller than that of Fe_NP_–N–C, which well explains the outstanding performance of Fe_SA_–N–C.

## Discussion

In summary, we have rationally developed a synthetic strategy toward high-loading SACs by nanocasting SiO_2_ into a MOF, PCN-222(Fe). The 3D skeleton of PCN-222(Fe) realizes the spatial isolation of Fe atoms that are bound by the N atoms in the linker. More importantly, the SiO_2_ nanocasted in the MOF mesochannels further offers anchoring effect, generating thermally stable FeN_4_/SiO_2_ interfaces and further inhibiting Fe agglomeration under pyrolysis. By integrating these dual protections, the SiO_2_@PCN-222(Fe) composite, as an ideal precursor, affords Fe_SA_–N–C with a Fe loading (3.46 wt%) higher than almost all reported single-atom Fe in N-doped carbon materials. Moreover, the synthetic approach is readily extendable to other single metal atoms, such as Co_SA_ and Ni_SA_. Thanks to the high-content Fe_SA_ sites, hierarchical pores and high conductivity of carbon matrix, Fe_SA_–N–C possesses excellent ORR performance in both alkaline and acidic media, far outperforming all other non-noble-metal catalysts and even the Pt/C. Furthermore, Fe_SA_–N–C delivers excellent performance in acidic PEMFC, demonstrating the great potential of Fe_SA_–N–C for PEMFC applications. We believe this nanocasting strategy might open up a fascinating avenue to the general fabrication of SACs with high loadings for broad applications.

## Methods

### Synthesis of PCN-222(Fe)

In a typical experiment, ZrOCl_2_ (108.6 mg), FeTCPPCl (32 mg), and CF_3_COOH (0.45 mL) were dissolved in DMF (10 mL), and ultrasonically dissolved in a 20 mL Pyrex vial. The mixture was heated in 120 °C oven for 18 h. After cooling down to room temperature, the obtained dark brown products were separated by centrifugation, and washed subsequently with DMF for thrice and acetone for twice. The as-obtained precipitates were activated in acetone and finally dried at 60 °C under vacuum overnight.

### Synthesis of SiO_2_@PCN-222(Fe)

Typically, PCN-222(Fe) (30 mg) was transferred to a two-necked flask and degassed for 12 h at 130 °C. When the system was cooled down to room temperature, TEOS (1200 μL) was injected into the flask, and the mixture was sonicated under vacuum for 20 min. Then the obtained solution was centrifuged and the solid was heated at 60 °C under vacuum for 20 min to remove the TEOS on the external surface of the MOF sample. After that, the sample was exposed to 3 M HCl vapor for 9 h at 60 °C to induce the polycondensation of TEOS within the mesopores of the MOF to give SiO_2_@PCN-222(Fe) composite.

### Synthesis of Fe_SA_–N–C

Typically, SiO_2_@PCN-222(Fe) was heated from the room temperature to 800 °C with a heating rate of 5 °C min^−1^, then maintained at this temperature for 2 h in N_2_ atmosphere. The metal oxides were removed by immersing the sample in the HF (20 wt%) solution for 6 h at 80 °C (Caution! HF is highly toxic and should be handled very carefully). The black sample was collected by centrifuging, washed several times with distilled water and ethanol, and dried at 60 °C under vacuum overnight.

### Synthesis of Fe_NP_–N–C

Typically, Fe_NP_–N–C was obtained from the direct pyrolysis of pure PCN-222(Fe) following the same procedure as Fe_SA_–N–C.

## Supplementary information

Supplementary Information

## Data Availability

The data that support the findings of this study are available from the corresponding author upon reasonable request.
