# Supplementary material for: Nanocasting SiO2 into metal–organic frameworks imparts dual protection to high-loading Fe single-atom electrocatalysts
Source: Nat Commun. 2020 Jun 5;11:2831. doi: 10.1038/s41467-020-16715-6 (PMC7275045; doi:10.1038/s41467-020-16715-6)
Supplement: Supplementary file 1 — Supplementary Information [file 41467_2020_16715_MOESM1_ESM.pdf]

Supplementary Information

Nanocasting SiO_2 into Metal-Organic Frameworks Imparts Dual Protection to High-Loading Fe Single-Atom Electrocatalysts

Jiao et al.

Supplementary Note 1 | Materials and Instrumentation.

All chemicals were purchased from commercial sources and used without further treatment: zirconyl chloride octahydrate ($ZrOCl_2 \cdot 8H_2O$, Aladdin Industrial Corporation, 98%), pyrrole (Aladdin Industrial Corporation, CP), iron(II) chloride tetrahydrate ($FeCl_2 \cdot 4H_2O$, Aladdin Industrial Corporation, 99%), cobalt(II) chloride hexahydrate ($CoCl_2 \cdot 6H_2O$, Sinopharm Chemical Reagent Co., Ltd., AR), nickel(II) chloride hexahydrate (Sinopharm Chemical Reagent Co., Ltd., AR), hydrofluoric acid (HF, Aladdin Industrial Corporation, 40%), anhydrous tetrahydrofuran (THF, Sinopharm Chemical Reagent Co., Ltd., 99.5%, anhydrous), ethanol (CH_3CH_2OH, Sinopharm Chemical Reagent Co., Ltd., 99.7%), N,N-dimethylformamide (DMF, Sinopharm Chemical Reagent Co., Ltd., AR), ethyl acetate (CH_3COOEt, Sinopharm Chemical Reagent Co., Ltd., AR), propanoic acid (C_2H_5COOH, Sinopharm Chemical Reagent Co., Ltd., AR), acetone (Sinopharm Chemical Reagent Co., Ltd., AR), hydrogen chloride (HCl, Sinopharm Chemical Reagent Co., Ltd., 37%), potassium hydroxide (KOH, Sinopharm Chemical Reagent Co., Ltd., 85%), trifluoroacetic acid (CF_3COOH, Energy Chemical, 99%), tetraethyl orthosilicate (TEOS, Energy Chemical, 98%), methyl 4-formylbenzoate (Energy Chemical, 99%). De-ionized water with the specific resistance of 18.25 MΩ·cm was obtained by reversed osmosis followed by ion-exchange and filtration (Cleaned Water Treatment Co., Ltd., Hefei).

Powder X-ray diffraction patterns (PXRD) were collected on a Japan Rigaku SmartLab™ rotation anode X-ray diffractometer equipped with graphite monochromatized Cu Kα radiation (λ = 1.54 Å). Fourier transform infrared (FT-IR)

spectra were measured using a Nicolet 8700 FTIR E.S.D. and KBr pellet samples. Elemental analysis (EA) was performed at VarioELIII analyzer. Raman scattering spectra were recorded with a Renishaw System 2000 spectrometer using the 514.5 nm line of Ar^+ for excitation. The transmission electron microscopy (TEM) and high-resolution TEM images were acquired on JEM-2010, and elemental mapping was measured on JEOL ARM-200F with an electron acceleration energy of 200 kV. Field-emission scanning electron microscopy (FE-SEM) was carried out with a field emission scanning electron microanalyzer (Zeiss Supra 40 scanning electron microscope at an acceleration voltage of 5 kV). Nitrogen sorption measurement was conducted using a Micromeritics ASAP 2020 system at 77 K. Prior to nitrogen adsorption/desorption measurement, the samples were dried overnight at 160 ℃ under vacuum. The Optima 7300 DV inductively coupled plasma atomic emission spectrometer (ICP-AES) was utilized for the quantification of the content of Fe. X-ray photoelectron spectroscopy (XPS) measurements were performed by an ESCALAB 250 high-performance electron spectrometer using monochromatized Al Kα (hv = 1486.7 eV) as the excitation source. The binding energy was calibrated using the C 1s from graphene oxide nanosheets located at 284.8 eV as the reference. The spectral decomposition was performed using the XPS Peak 41 program with Gaussian functions after subtraction of a Shirley background.

Supplementary Note 2 | X-ray Adsorption Spectra.

The Fe/Co/Ni K-edge X-ray absorption near edge structure (XANES) and extended X-ray absorption fine structure (EXAFS) experiments were carried out at the beamlines 5BM-D and 20BM-B of Advanced Photon Source (APS) at Argonne National Laboratory (ANL). The catalyst powder and reference were pressed into pellets with boron nitrides and measured in transmission mode. The incident beam was monochromatized by using a Si (111) fixed-exit, double-crystal monochromator, and a harmonic rejection mirror was applied to cut off the harmonics at higher X-ray energy. Data reduction, data analysis, and EXAFS fitting were performed with the Athena and Artemiss software packages. The energy calibration of the catalysts was conducted through a standard metal foil, which as a reference was simultaneously measured.

Data points were acquired in three separate regions (energies relative to the Fe edges): a pre-edge region (-300 to -30 eV, step size of 10 eV), the XANES region (-30 to +30 eV, step size of 0.5 eV) and the EXAFS region (to 12.5 $Å^{-1}$, step size of 0.07 $Å^{-1}$, dwell time of 1 s). The ionization chambers were optimized for the maximum current with a linear response (~1010 photons detected per second) with 10% absorption in the incident ion chamber and 70% absorption in the transmission detector.

Standard procedures for the normalization and background subtraction were performed using the Demeter 0.9.25 software package. XANES at Fe K-edge was determined from the inflection point in the leading edge, that is, the maximum in the

first derivative of the leading edge of the XANES spectrum. The pre-edge energies were also obtained in the first derivative using the zero-crossing point.

For EXAFS modeling, EXAFS of the Fe foil is fitted and the obtained amplitude reduction factor S_0^2 value (0.81) was set in the EXAFS analysis to determine the coordination numbers (CNs) in the Fe-N scattering path in Fe_{SA}-N-C catalysts.

Following the standard EXAFS fitting procedures and model construction (the model of Fe-N_4-C as is shown in Figure 3c) that have been demonstrated in the previous studies[1-2], the EXAFS fitting was conducted through a least-squares fitting in R space for the k^2-weighted Fourier transformed data using Artemis.

The obtained best fitting results can be found in Supplementary Table 4, all the parameters are found within the reasonable ranges, indicating a good match between the model and the experimental data. Additionally, this can be also evidenced by the good match between the k-space oscillations the experimentally data and the as-generated from the model (Supplementary Figure 14).

Supplementary Note 3 | Ligand Synthesis.

The ligand was synthesized according to the previous report with modifications.[3-4] The typical synthetic procedures are described below.

5,10,15,20-Tetrakis(4-methoxycarbonylphenyl)porphyrin (TPPCOOMe).

Typically, pyrrole (3.0 g, 0.043 mol), methyl 4-formylbenzoate (6.9 g, 0.042 mol) and propionic acid (100 mL) were added to a 500 mL three-necked flask to form mixed solution, which was then refluxed at 140 ℃ for 12 h in darkness. After the reaction mixture was cooled down to room temperature, purple crystals were collected by suction-filtration. Then the crystals were washed in the sequence of ethanol, ethyl acetate and THF, and the obtained crystals were dried under vacuum at 60 ℃.

[5,10,15,20-Tetrakis(4-methoxycarbonylphenyl)porphyrinato]-Fe(III) Chloride (Fe-TPPCOOMe), Co-TPPCOOMe and Ni-TPPCOOMe.

Typically, TPP-COOMe (0.854 g, 1.0 mmol) was dissolved in DMF solution (100 mL) containing $FeCl_2•4H_2O$ (2.5 g, 12.8 mmol) and the mixed solution was refluxed at 160 ℃ for 6 h. When the mixture was cooled down to room temperature, 150 mL of H_2O was added. The resultant precipitate was filtered and washed with 50 mL of H_2O for twice. The obtained solid was dissolved in $CHCl_3$, followed by extracting three times with 1 M HCl and twice with water. The Co-TPPCOOMe and Ni-TPPCOOMe were synthesized following the same procedure as Fe-TCPPCl, except for $CoCl_2•6H_2O$ (3.1 g, 12.8 mmol) and $NiCl_2•6H_2O$ (3.1 g, 12.8 mmol) were used instead of $FeCl_2•4H_2O$ (2.5 g, 12.8 mmol) to synthesis Co-TPPCOOMe and Ni-TPPCOOMe.

[5,10,15,20-Tetrakis(4-carboxyphenyl)porphyrinato]-Fe(III) Chloride (Fe-TCPPCl), Co-TCPP, and Ni-TCPP.

The obtained Fe-TPPCOOMe (0.75 g), Co-TPPCOOMe (0.75 g) and Ni-TPPCOOMe (0.75 g), was dissolved in the mixed solvent of THF (25 mL) and MeOH (25 mL), to which 25 mL aqueous solution of KOH (2.63 g, 46.95 mmol) was introduced. The obtained mixture was refluxed at 85 ℃ for 12 h. After cooling down to room temperature, THF and MeOH were evaporated. Additional water was added to the resulting water phase and the mixture was heated until the solid was fully dissolved, then the homogeneous solution was gradually acidified with 1 M HCl until no further precipitate was detected. The brown solid was collected by filtration, washed with water and dried in vacuum.

Tetrakis(4-carboxyphenyl)porphyrin (H_2-TCPP)

Firstly, the obtained ester TPPCOOMe (0.75 g) was stirred in THF (25 mL) and MeOH (25 mL) mixed solvent, to which a solution of KOH (2.63 g, 46.95 mmol) containing 25 mL H_2O was introduced. Next, the mixture was refluxed at 85 ℃ for 12 h. After cooling down to room temperature, the mixture was transferred to a beaker, to which 1 M HCl was gradually added until pH reached 3~4, accompanied by the formation of precipitate. At length, the obtained product was washed with water for three times and dried under vacuum at 60 ℃.

Supplementary Note 4 | Synthesis of PCN-222(Co) and PCN-222(Ni).

In a typical experiment, $ZrOCl_4$ (108.6 mg), Co-TCPP (31 mg) or Ni-TCPP (31 mg), and CF_3COOH (0.45 mL) were dissolved in DMF (10 mL) and ultrasonically dissolved in a 20 mL Pyrex vial. The mixture was heated in 120 ℃ oven for 18 h. After cooling down to room temperature, the obtained dark brown products were separated by centrifugation and washed subsequently with DMF for thrice and acetone for twice. The as-obtained precipitates were activated in acetone and finally dried at 60 ℃ under vacuum overnight.

Supplementary Note 5 | Synthesis of PCN-222.

The PCN-222 was synthesized following the same procedure as PCN-222(Fe), except for the addition of 30 mg H_2-TCPP ligand (in the absence of Fe center in the porphyrin linker) instead of 32 mg Fe-TCPPCl.

Supplementary Note 6 | Synthesis of SiO_2@PCN-222, SiO_2@PCN-222(Co) and SiO_2@PCN-222(Ni) composites.

The procedure is similar to that for SiO_2@PCN-222(Fe) described above, except for PCN-222 (30 mg), PCN-222(Co) (30 mg) and PCN-222(Ni) (30 mg) were used instead of PCN-222(Fe) (30 mg) to synthesis SiO_2@PCN-222, SiO_2@PCN-222(Co) and SiO_2@PCN-222(Ni).

Supplementary Note 7 | Synthesis of N-C catalysts.

Typically, the N-C was synthesized through the similar methods to Fe_{SA}-N-C except for employing SiO_2@PCN-222 as the precursor.

Supplementary Note 8 | Synthesis of Co_{SA}-N-C and Ni_{SA}-N-C catalysts.

The Co_{SA}-N-C and Ni_{SA}-N-C were synthesized through the similar methods to Fe_{SA}-N-C except for employing SiO_2@PCN-222(Co) and SiO_2@PCN-222(Ni) as precursors, respectively.

Supplementary Note 9 | Electrochemical measurements.

Electrochemical measurements were performed with a CHI 760E electrochemical analyzer (CH Instruments, Inc., Shanghai) and a rotating disk electrode (RDE) (Pine Instruments, Grove city, PA). All electrochemical measurements were conducted in a typical three-electrode setup with a graphite rod counter electrode and Ag/AgCl reference electrode. LSV measurements were conducted with scan rate of 5 mV/s. All potentials reported in this work were converted from vs Ag/AgCl to vs RHE by adding a value of 0.197 + 0.059 × pH. All data are presented without iR compensation.

The catalyst ink was prepared by dispersing 2 mg of catalyst into 1 mL of ethanol containing 10 μL of 5 wt% Nafion and sonicated for 30 min. Then 28 μL of the catalyst ink was loaded onto a GCE of 5 mm diameter (loading amount: ~0.28 mg cm^{-2}). For comparison, Pt/C (20 wt% platinum) was conducted on the same electrochemical tests with a catalyst loading of 0.1 mg cm^{-2}.

For the ORR at a RDE, the electron transfer number can be calculated with Koutecky-Levich equations:

$$\frac{1}{j} = \frac{1}{j_L} + \frac{1}{j_K} = \frac{1}{B\omega^{1/2}} + \frac{1}{j_K} \qquad (1)$$

$$B = 0.62nFC_0(D_0)^{2/3}\nu^{-1} \qquad (2)$$

$$j_K = nFkC_0 \qquad (3)$$

where j is the measured current density; j_k and j_L are the kinetic and diffusion-limiting current densities, respectively; ω is the angular velocity of the disk (= 2πN, N is the linear rotation speed); *n* represents the overall number of electrons

transferred in oxygen reduction; F is the Faraday constant (F = 96485 C mol^{-1}); C_0 is the bulk concentration of O_2 (1.2×10^{-6} mol cm^{-3}); D_0 is the diffusion coefficient of O_2 in 0.1 M KOH electrolyte (1.9×10^{-5} cm^2 s^{-1}); v is the kinematics viscosity for electrolyte, and k is the electron-transferred rate constant.

Rotating ring-disk electrode (RRDE) measurements were carried out to determine the four-electron selectivity. The disk electrode was scanned at a rate of 10 mV s^{-1}, and the ring electrode potential was set to 1.2 V *vs.* RHE. The hydrogen peroxide yield (%H_2O_2) and the electron transfer number (n) were calculated by the following equations:

$$\%H_2O_2 = 200\frac{i_r/N}{i_d + i_r/N} \qquad (4)$$

$$n = 4\frac{i_d}{i_d + i_r/N} \qquad (5)$$

where i_d and i_r are the disk and ring currents, respectively. The N represents the ring current collection efficiency which was determined to be ~37 %.

Supplementary Note 10 | PEMFC tests.

The catalyst was mixed with Nafion® alcohol solution (5 wt%, Aldrich), isopropanol and deionized water to prepare the catalyst ink, which contained the same weight of Nafion ionomer as the catalyst. The ink was subjected to a sonication for 10 min and a stirring for 12 h. The well-dispersed ink was brushed on a piece of carbon paper (5 cm^2), followed by a drying in vacuum at 80 ℃ for 2 h. The prepared cathode and anode were pressed onto the two sides of a Nafion 211 membrane (DuPont) at 130 ℃ for 90 seconds under a pressure of 1.5 MPa to obtain the membrane electrode assembly (MEA). Polarization plots were recorded using fuel cell test station (Scribner 850e) in a current-scanning mode.

Test conditions: cathode loading 3.0 mg cm^{-2} for Fe_{SA}-N-C or 0.2 mg_{Pt} cm^{-2} for Pt/C, anode loading 0.2 mg_{Pt} cm^{-2}, Nafion 211 membrane, 5 cm^2 electrode area, 80 ℃, 100% RH, 2 bar O_2/H_2. UHP-grade H_2 and O_2 humidified at 80 ℃ were supplied to the anode and cathode at a flow rate of 0.3 and 0.4 L min^{-1}, respectively. The backpressures at both electrodes were set at 1.5 bar.

After recording the polarization curves, a 20 h stability test was performed on the same MEA, while the flow rate of the gas was switch to 0.1 L min^{-1} for both electrodes.

Supplementary Note 11 | Computational Details.

The stabilization effect of SiO_2 for single-atom Fe.

The calculations were performed with the VASP 5.4.1 program. The Perdew-Burke-Ernzerhof (PBE) functional and projector augmented wave (PAW) potentials were adopted to complete the DFT calculations. In detail, the spin polarization correction was included to consider the magnetism effect. A 4×4 graphene with vacuum layer of 15 Å was used to simulate the catalyst surface. The calculation software was Vienna ab initio simulation package. Considering the calculation accuracy and cost, 3×3×1 Γ-centered k-point and 500 eV cutoff energy were adopted. The convergence standards of energy and force were selected as 10^{-5} eV and 0.05 eV $Å^{-1}$. The vibrational frequency with finite displacements of ± 0.02 Å was calculated to guarantee only one imaginary frequency in transition state structures.

ORR reaction mechanism of Fe_{SA}-N-C.

The geometry optimization and electronic properties of the periodic structures are performed using the projector augmented wave (PAW) method implemented in the Vienna ab initio simulation package (VASP) code.[5-8] The generalized gradient approximation (GGA) functional to the exchange-correction functional, of Perdew-Burke-Ernzerhof (PBE) form with vdW corrections (VDW-DFT) is adopted in our calculations. The cut-off energy is set to 450 eV. To module the Fe_{SA}-N-C electrocatalyst, a Fe-N_4 site is embedded in a periodic 4*4 graphene support (32 carbon sites) with lattice parameters a = b = 9.87 Å and γ = 60°. The vacuum spacing

is set to more than 15 Å along the surface normal to avoid the interactions between images. Gamma centred 3*3*1 k-points grid is chosen for relaxations and property calculations. In this calculation, the convergences of energy and force are selected as 0.01 eV $Å^{-1}$ and 10^{-4} ev, respectively.

The overall ORR process can be divided into four elementary steps (* in the following equations indicates the active sites):

(i) $O_2 + H^+ + e^- + * = OOH*$

(ii) $OOH* + H^+ + e^- = O* + H_2O$

(iii) $O* + H^+ + e^- = OH*$

(iv) $OH* + H^+ + e^- = H_2O$

Following the methodology developed by Norskov et al,[9] the chemical potential (the free energy per H) for the reaction ($H^+ + e^-$) is equal to that of 1/2 H_2 by setting the reference potential to be that of the standard hydrogen electrode at standard condition (pH = 0, P_{H2} = 1 bar, and T = 298 K). As a result, the reaction free energy (ΔG) is further calculated by the following formula:

$$\Delta G = \Delta H - T\Delta S - qU + k_BT\ln10*(pH) \quad (6)$$

Where ΔH is the reaction enthalpy of an elementary step in ORR and is estimated by the reaction energy (ΔE) from DFT calculations with zero-point energy (ZPE) correction; TΔS is the change in entropy contribution to the free energy; U is the applied potential; q is the charge transfer in each elementary step. Note that the energy (i.e. enthalpy) of $H_2O(l)$ is approximately by that of $H_2O(g)$, while the entropy of $H_2O(l)$ is calculated by $S_{H2O(g)} + \Delta S_{g\text{-}l}$, where the entropy change from $H_2O(g)$ to

$H_2O(l)$, $S_{g\text{-}l}$ is chosen to be -118.9 J K^{-1} mol^{-1} at 298 K, obtained from the "CRC Handbook of Chemistry and Physics".[10]

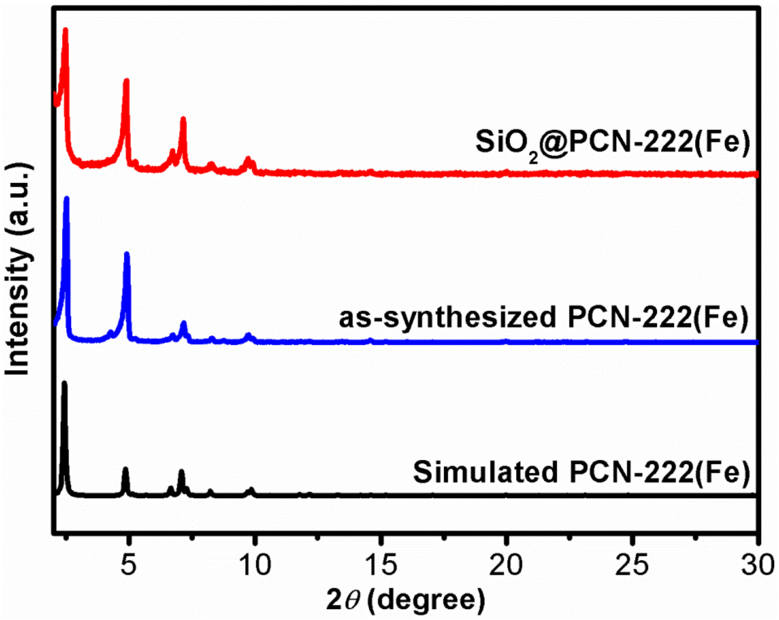

Supplementary Figure 1 | Powder XRD patterns of PCN-222(Fe) and SiO_2@PCN-222(Fe).

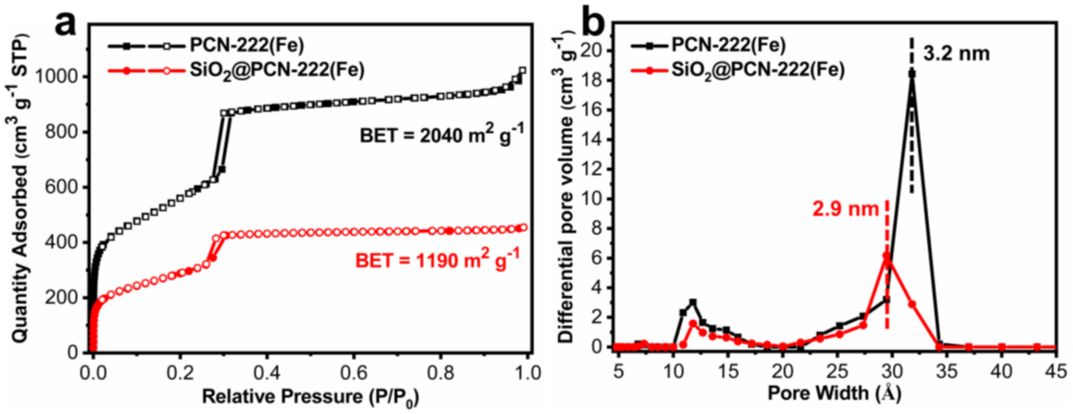

Supplementary Figure 2 | N_2 sorption isotherms and pore-size distribution curves for PCN-222(Fe). (a) N_2 sorption isotherms at 77 K and (b) the corresponding pore size distributions calculated by the DFT model for PCN-222(Fe) and SiO_2@PCN-222(Fe).

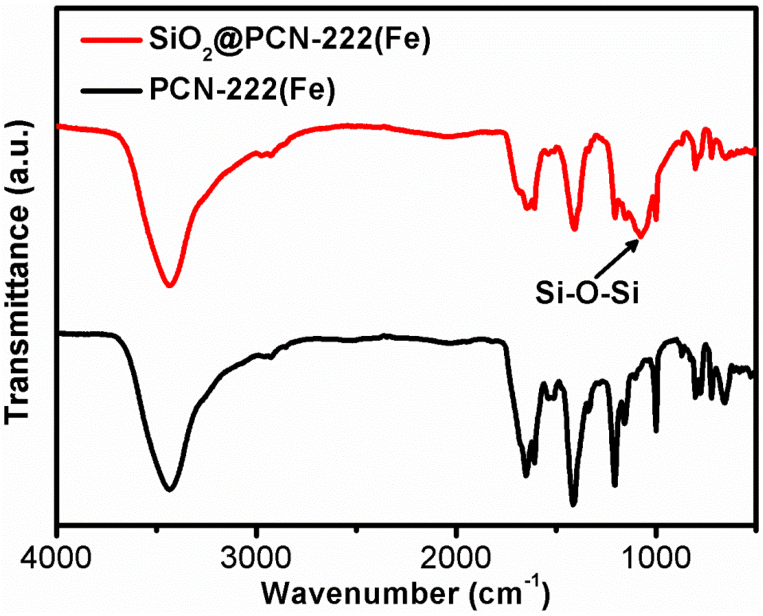

Supplementary Figure 3 | FT-IR spectra. FT-IR spectra of PCN-222(Fe) and SiO_2@PCN-222(Fe). The presence of silica in the SiO_2@PCN-222(Fe) is confirmed by the Si-O-Si absorption peak at 1090 cm^{-1}.

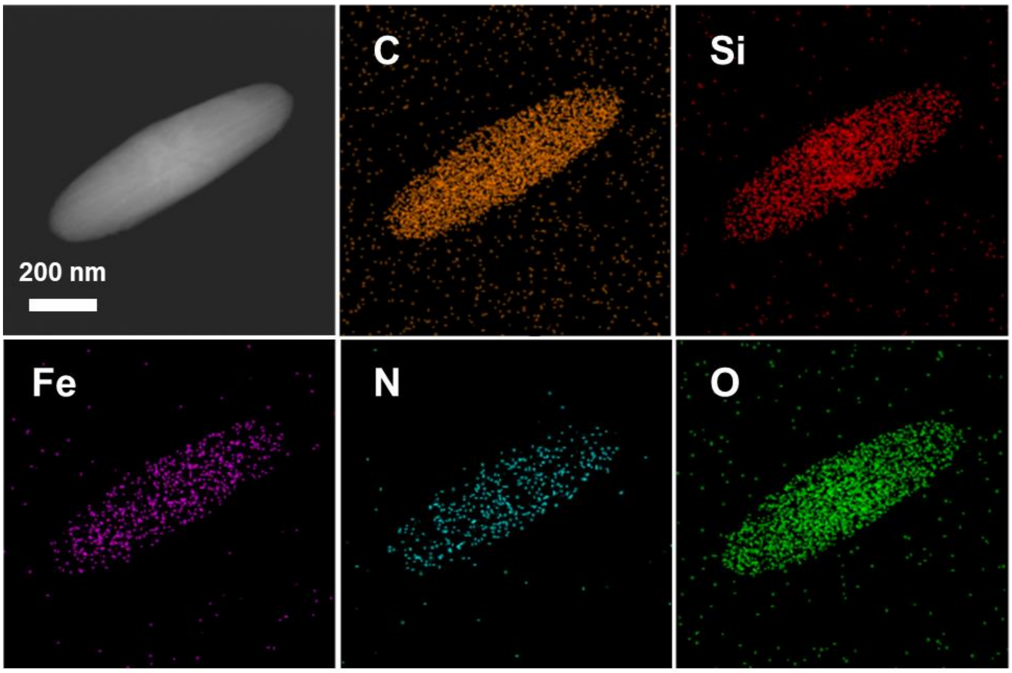

Supplementary Figure 4 | Elemental mapping images. The HAADF-STEM and related mapping images for C, Si, Fe, N and O elements in SiO_2@PCN-222(Fe).

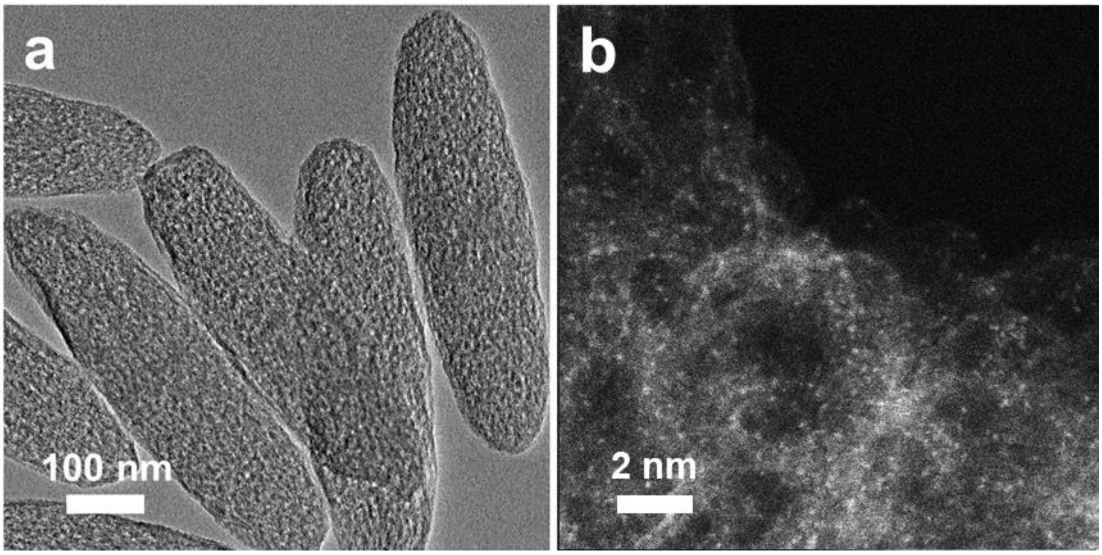

Supplementary Figure 5 | TEM and HAADF-STEM images. (a) TEM image and (b) aberration-corrected HAADF-STEM image of Fe_{SA}-N-C.

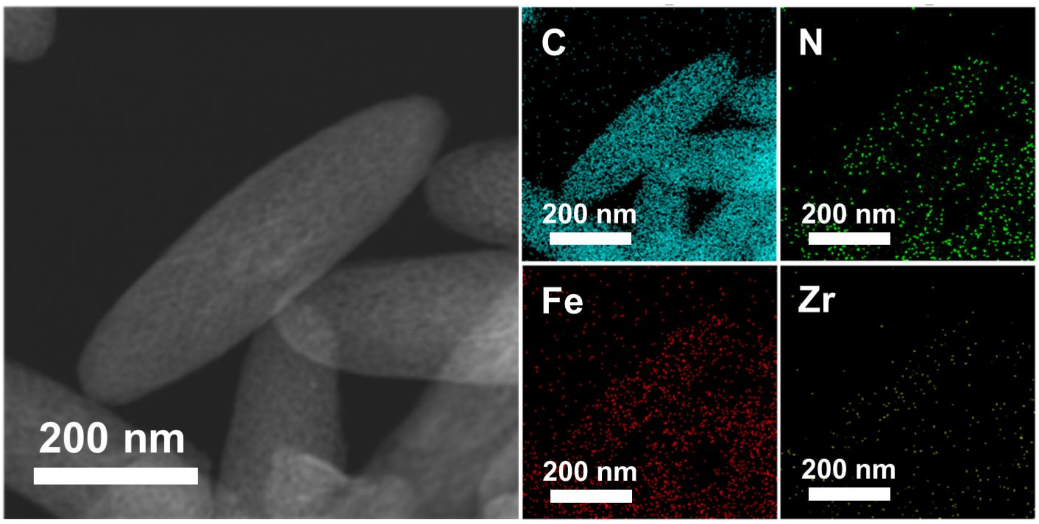

Supplementary Figure 6 | Elemental mapping images. The HAADF-STEM and elemental mapping images of Fe_{SA}-N-C. The images clearly demonstrate the homogeneous dispersion of C, N and Fe. The signal of Zr is relatively weak compared with Fe manifesting its much lower content than Fe in Fe_{SA}-N-C.

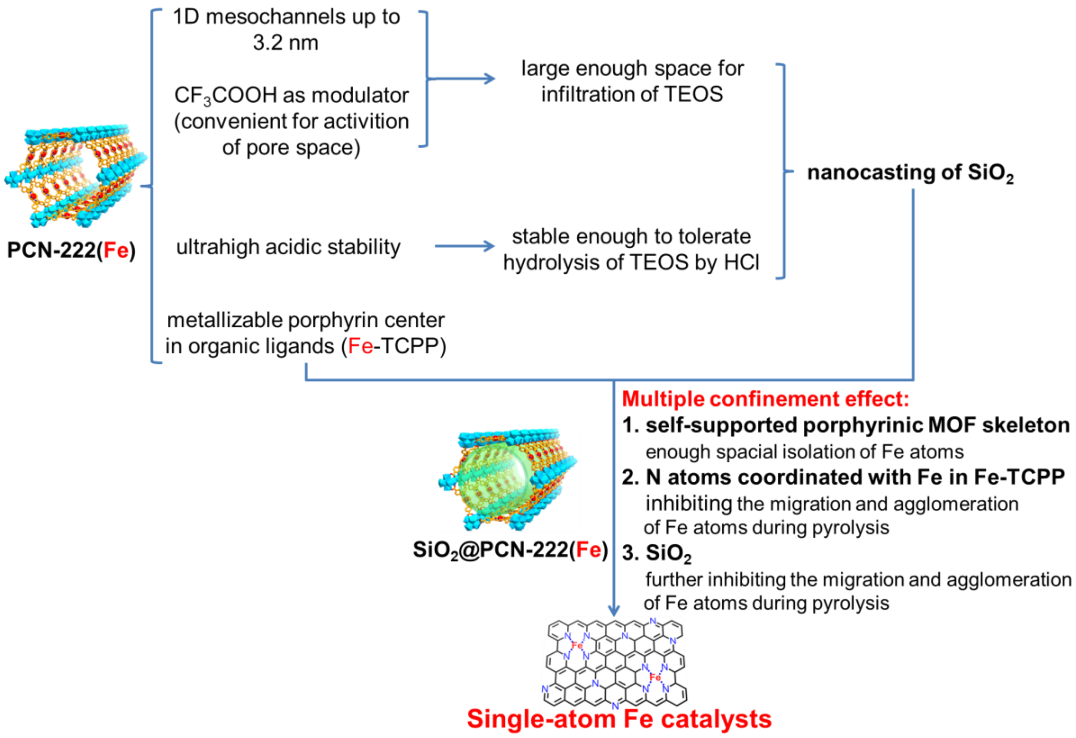

Supplementary Figure 7 | Illustration for the fabrication of single-atom Fe catalysts. Design for the nanocasting-assisted construction of high-content single-atom Fe catalysts from PCN-222(Fe).

A highly stable MOF involving organic linkers with metallizable porphyrin centers and 1D mesochannels up to 3.2 nm, is selected as starting template material for nanocasting. The porphyrin centers create conditions for the incorporation of targeted single metal atoms, and the 1D mesochannels make it feasible to achieve the full filling of guests during nanocasting process. Meanwhile, the very high stability of PCN-222(Fe) in acidic media makes it possible to induce the condensation of TEOS by HCl without framework collapse. Intergrating the merits of both SiO_2 and PCN-222(Fe), the resulting SiO_2@PCN-222(Fe) composite affords an ideal precursor to create atomically dispersed Fe sites in high loadings in MOF-derived porous carbons after high-temperature pyrolysis, by virtue of the interaction and stabilization effect with N atoms and SiO_2, respectively.

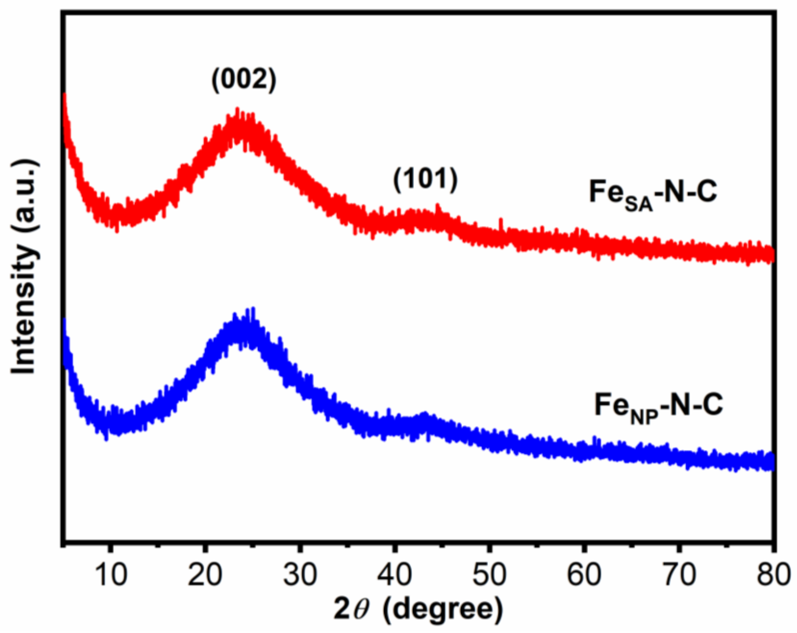

Supplementary Figure 8 | PXRD pattern. PXRD patterns of Fe_{SA}-N-C and Fe_{NP}-N-C.

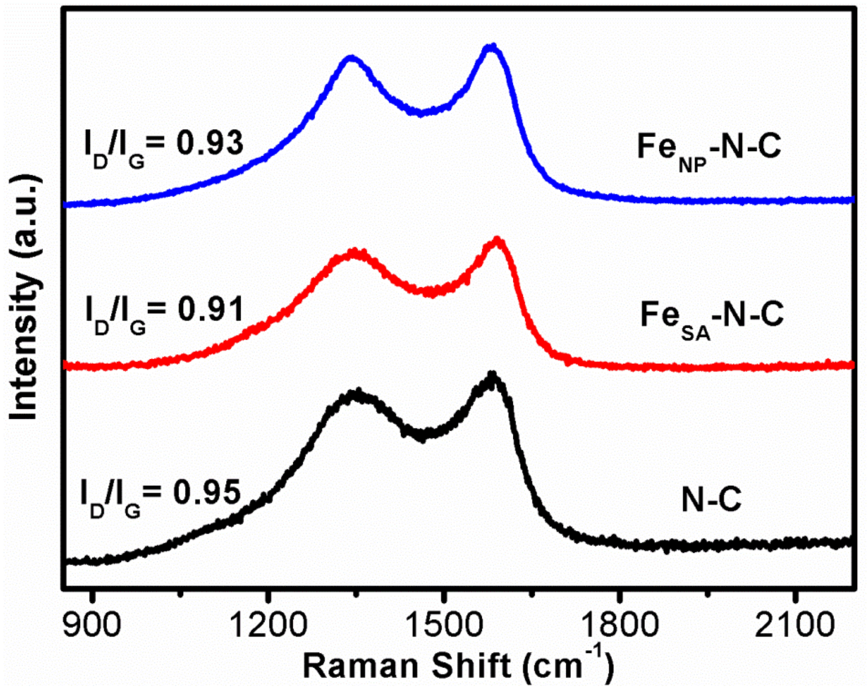

Supplementary Figure 9 | Raman spectra. Raman spectra of N-C, Fe_{SA}-N-C and Fe_{NP}-N-C.

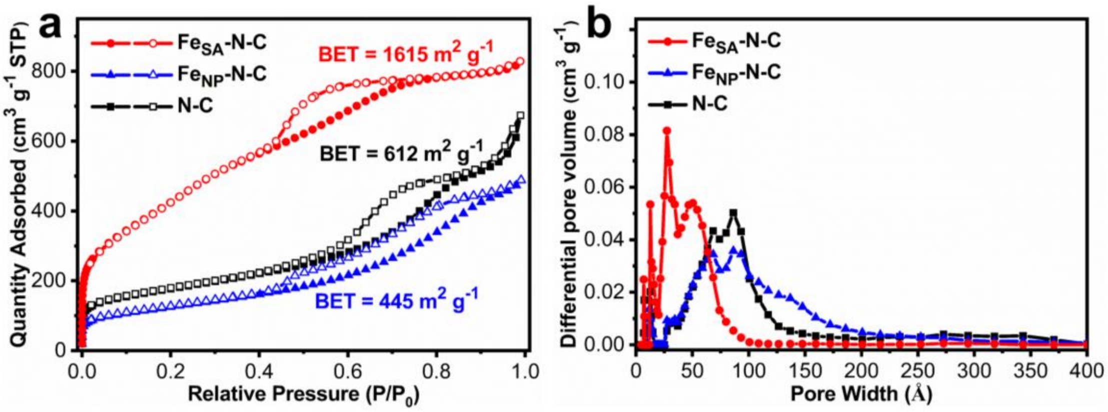

Supplementary Figure 10 | N_2 sorption isotherm and pore-size distribution curves. (a) N_2 sorption isotherm at 77 K of N-C, Fe_{SA}-N-C and Fe_{NP}-N-C and (b) the corresponding pore size distribution calculated by the DFT model.

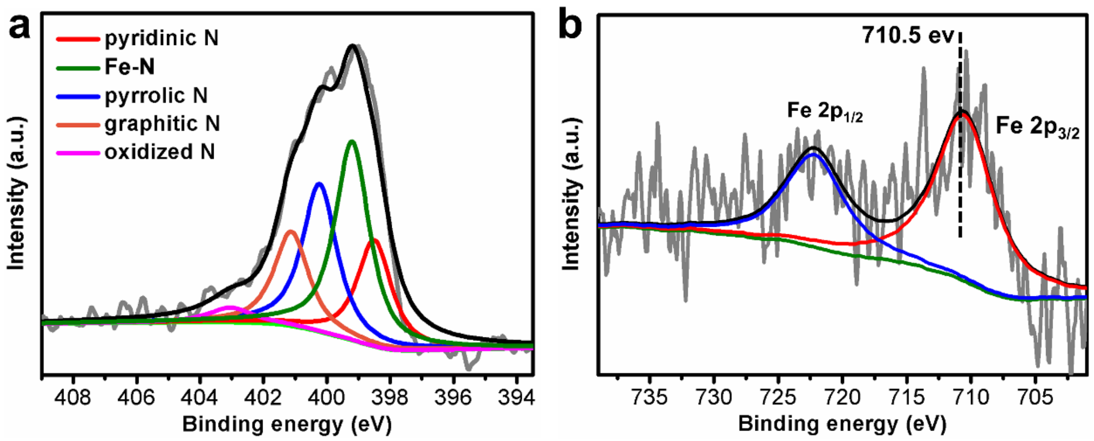

Supplementary Figure 11 | XPS spectra. The (a) N 1s and (b) Fe 2p spectra of Fe_{SA}-N-C.

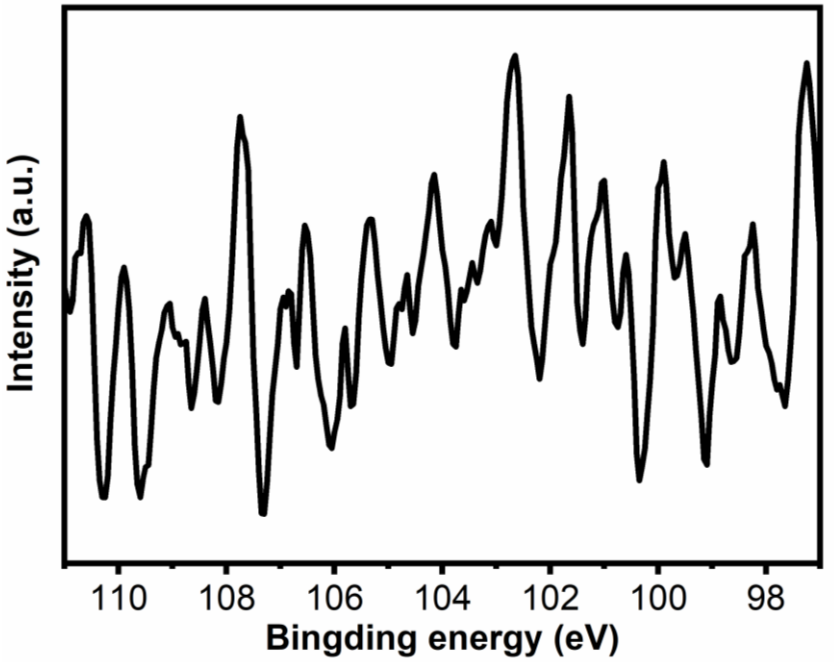

Supplementary Figure 12 | XPS spectrum. The Si 2p XPS spectrum. The unobservable Si signal indicates the Si residual is negligible in the Fe_{SA}-N-C.

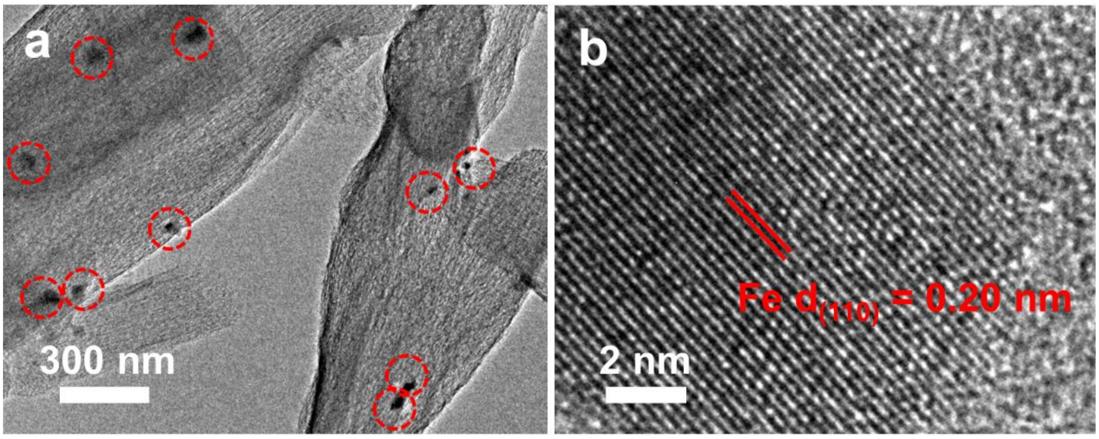

Supplementary Figure 13 | TEM images. (a) TEM and (b) HRTEM images of Fe_{NP}-N-C.

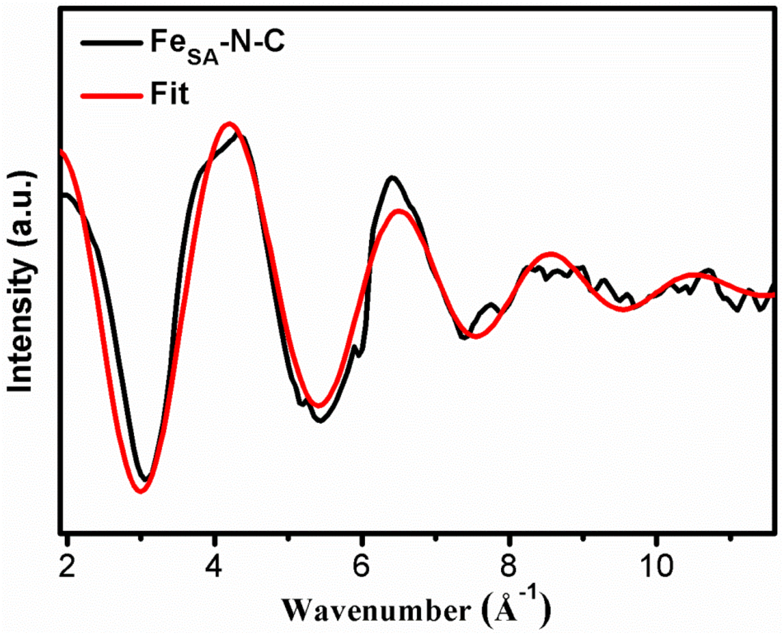

Supplementary Figure 14 | The corresponding EXAFS *k*-space fitting results of Fe$_{SA}$-N-C.

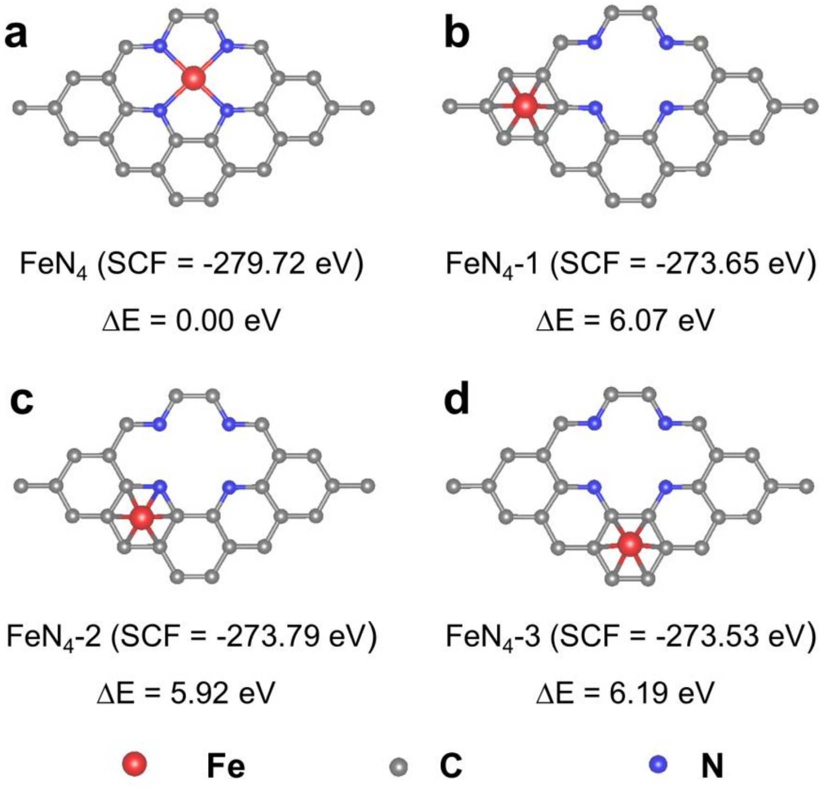

Supplementary Figure 15 | The model of FeN$_4$ and migration structures. (a) The structure of FeN$_4$, (b)-(d) Three possible migration structures.

To investigate the migration of Fe atom on the N-doped carbon surface, we considered different migration structures and obtained three stable migration structures, as shown in Figure S15. In Figure S15, the ground state energies of the corresponding systems were labelled. To compare the energy differences between different structures, the energy of FeN_4 was selected as the zero of relative energy. To be specific, the relative energy of FeN_4-2 is smallest, with a value of 5.92 eV, indicating that the stable structure of FeN_4 is inclined to form the structure of FeN_4-2 among three possible migration structures in the view of thermodynamics. Therefore, the structure of FeN_4-2 was selected as the migratory structure in the subsequent migration calculations of Fe atom.

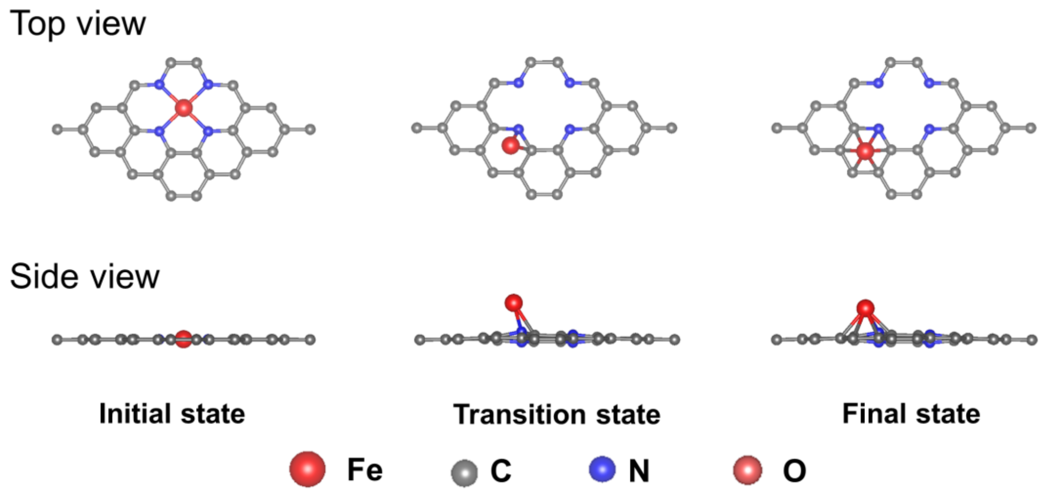

Supplementary Figure 16 | Configurations of FeN_4 during the migration process.

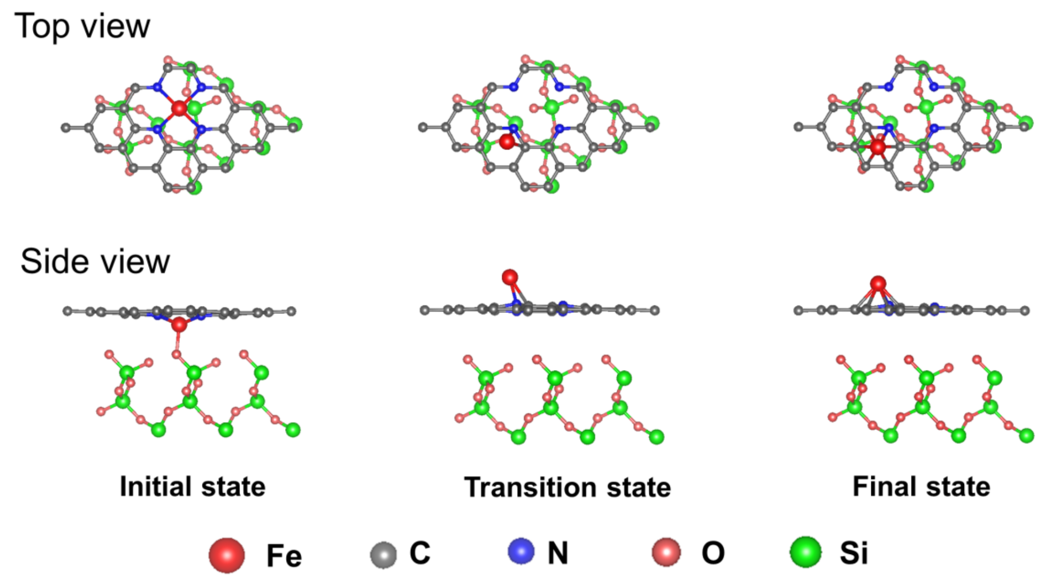

Supplementary Figure 17 | Configurations of FeN_4/SiO_2 during the migration process.

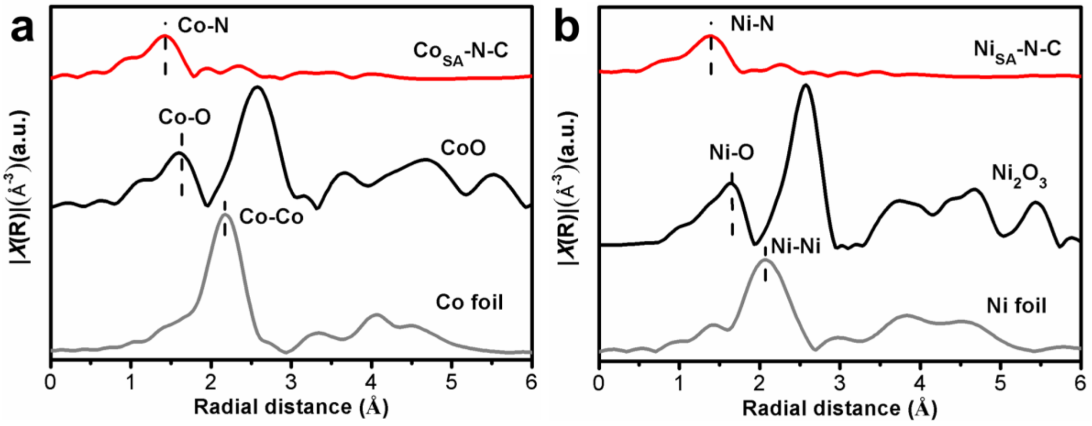

Supplementary Figure 18 | Fourier transform-extended X-ray asorption fine structure (FT-EXAFS) spectra. FT-EXAFS spectra of (a) Co_{SA}-N-C and (b) Ni_{SA}-N-C.

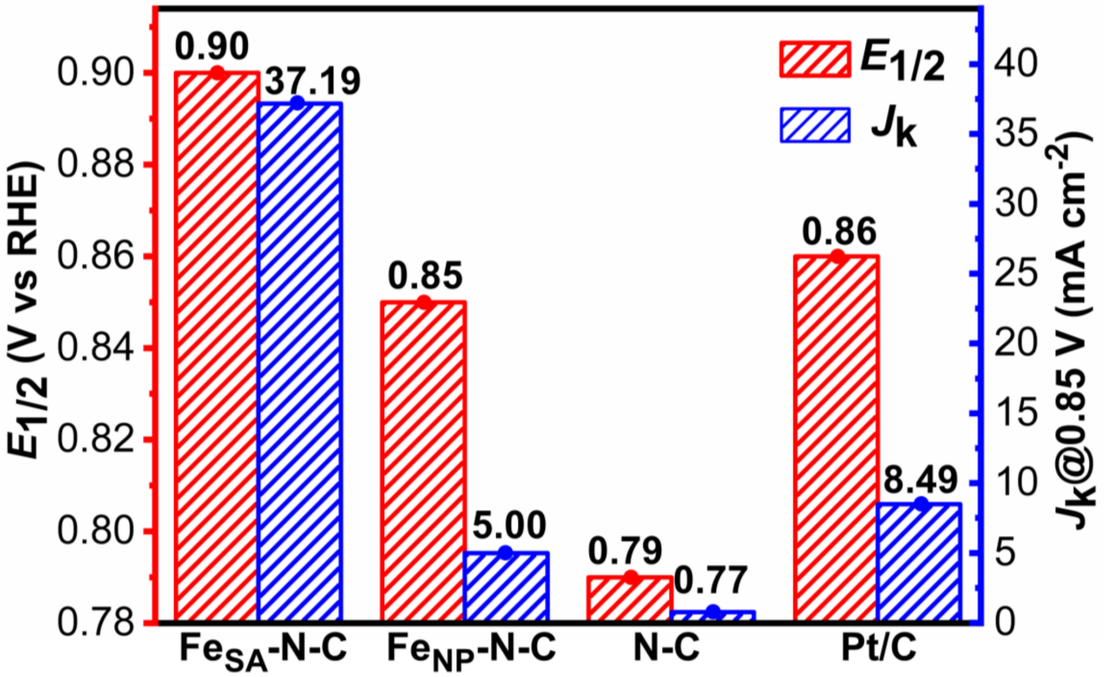

Supplementary Figure 19 | Activity comparison. Comparison of $E_{1/2}$ and J_k at 0.85 V for various catalysts in 0.1 M KOH.

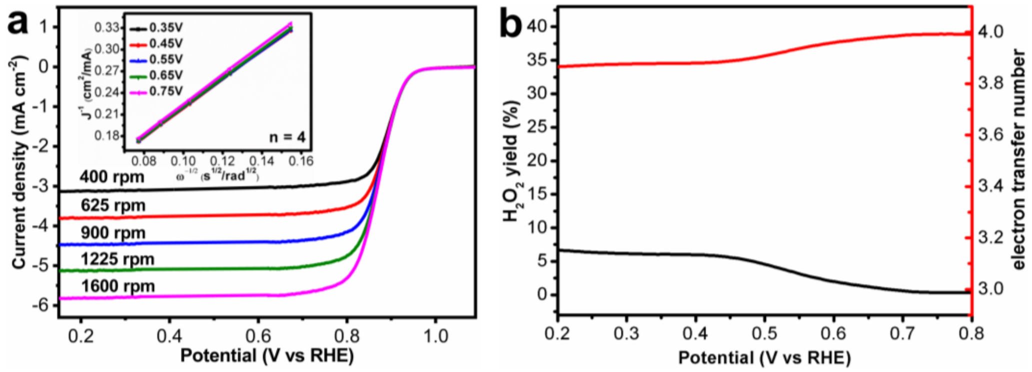

Supplementary Figure 20 | Selectivity characterizations of Fe_{SA}-N-C in 0.1 M KOH. (a) LSV curves of Fe_{SA}-N-C at different rotating rates in 0.1 M KOH (inset in a K-L plots and electron transfer number). (b) Peroxide yield and the calculated electron transfer number of Fe_{SA}-N-C in 0.1 M KOH.

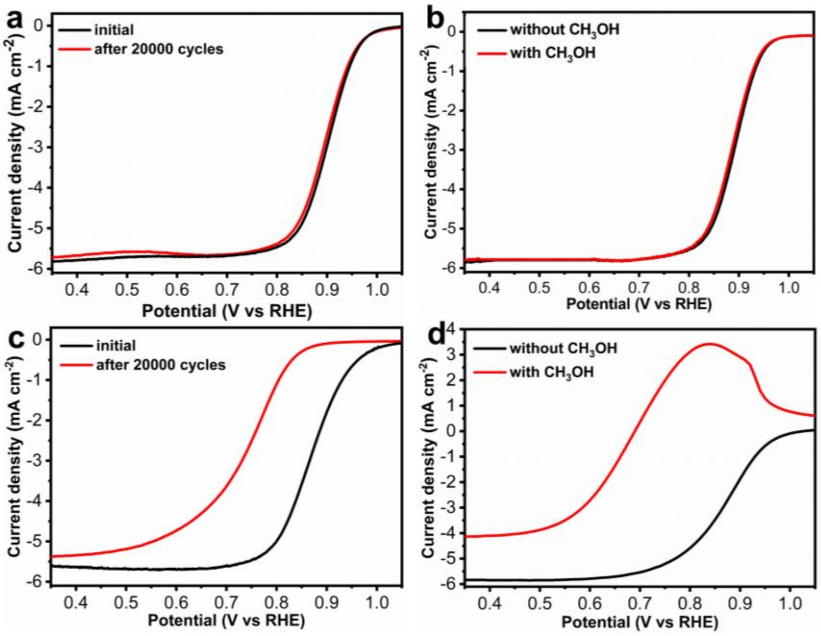

Supplementary Figure 21 | Stability of Fe_{SA}-N-C and Pt/C in 0.1 M KOH solution. LSV curves of (a, b) Fe_{SA}-N-C and (c, d) the commercial Pt/C (a, c) before and after 20000 cycles and (b, d) before and after the addition of CH_3OH in O_2-saturated 0.1 M KOH solution.

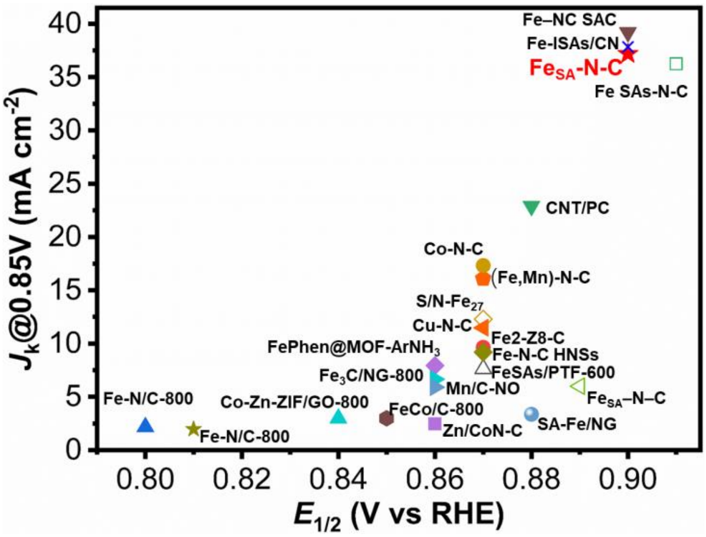

Supplementary Figure 22 | ORR activity comparison in alkaline media. Comparison of ORR performance between Fe_{SA}-N-C and other non-noble metal electrocatalysts in 0.1 M KOH solution.

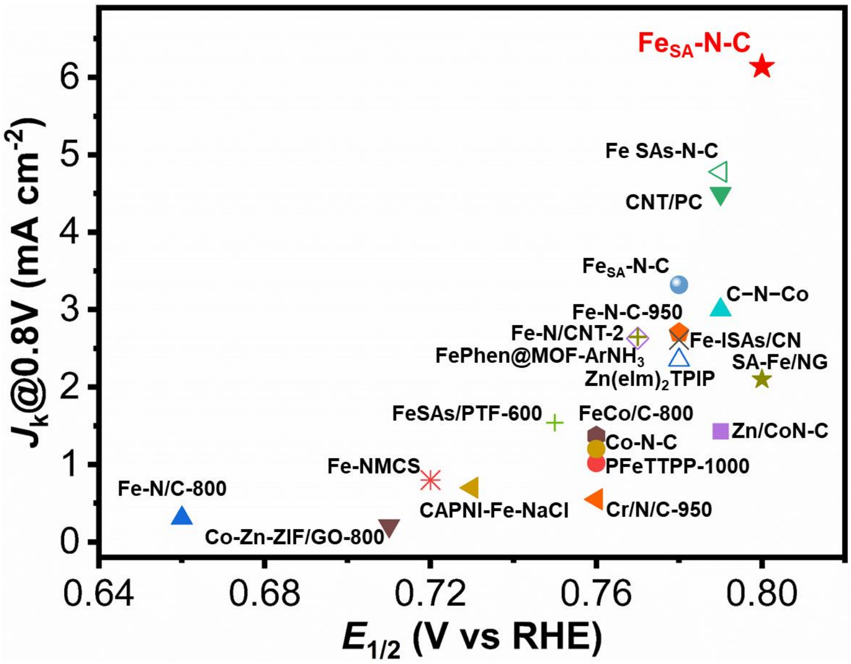

Supplementary Figure 23 | ORR activity comparison in acidic media. Comparison of ORR performance between Fe$_{SA}$-N-C and other non-noble metal electrocatalysts in 0.1 M $HClO_4$ solution.

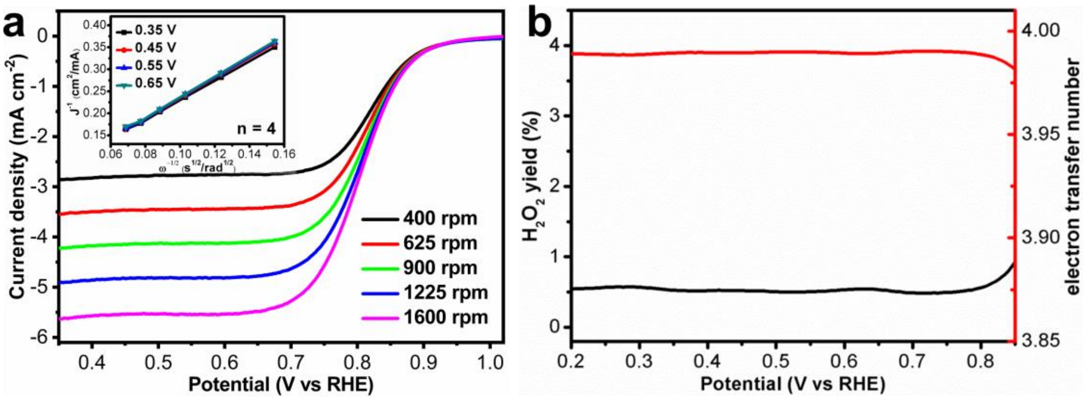

Supplementary Figure 24 | Selectivity characterizations of Fe$_{SA}$-N-C in 0.1 M $HClO_4$. (a) LSV curves at different rotating rates (inset in c: K-L plots and electron transfer number) and (b) peroxide yield and the calculated electron transfer number of Fe$_{SA}$-N-C in 0.1 M $HClO_4$.

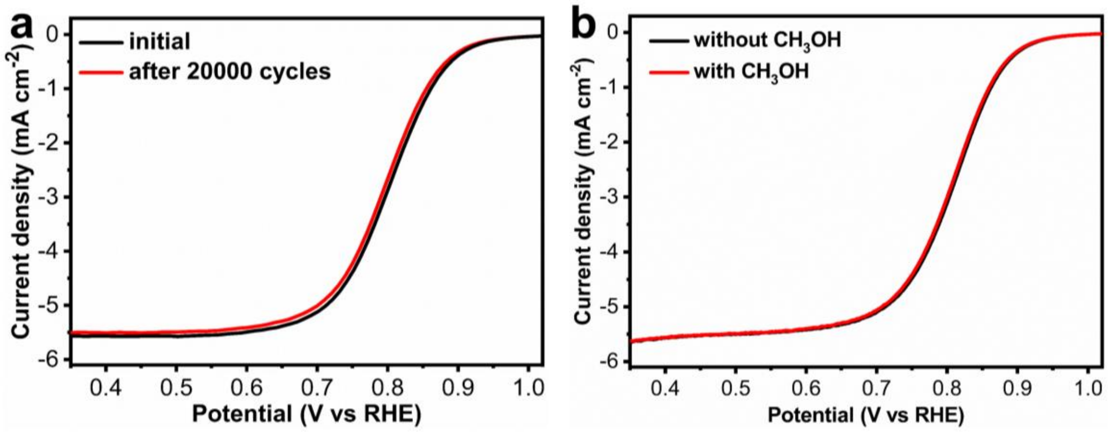

Supplementary Figure 25 | Stability of Fe_{SA}-N-C in 0.1 M $HClO_4$ solution. LSV curves of Fe_{SA}-N-C (a) before and after 20000 cycles and (b) before and after the addition of CH_3OH in O_2-saturated 0.1 M $HClO_4$ solution.

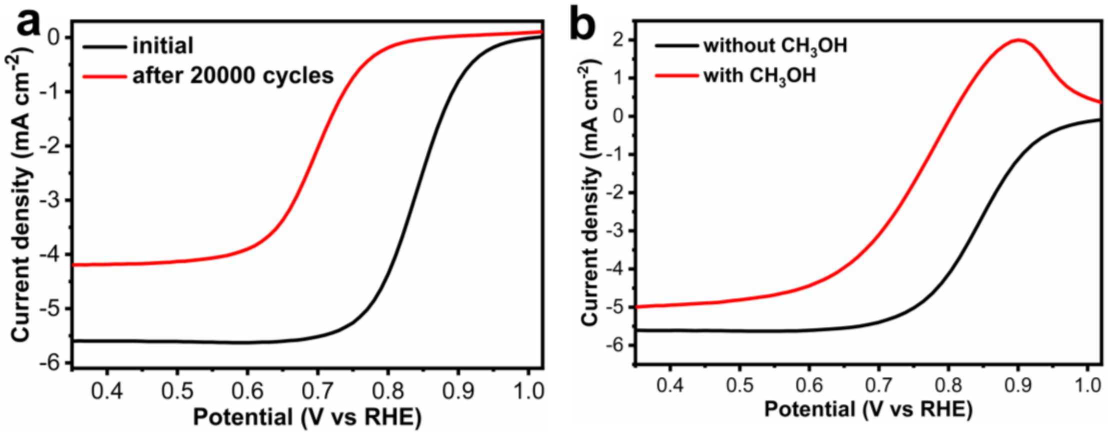

Supplementary Figure 26 | Stability of Pt/C in 0.1 M $HClO_4$ solution. LSV curves of commercial Pt/C (a) before and after 20000 cycles and (b) before and after the addition of CH_3OH in O_2-saturated 0.1 M $HClO_4$ solution.

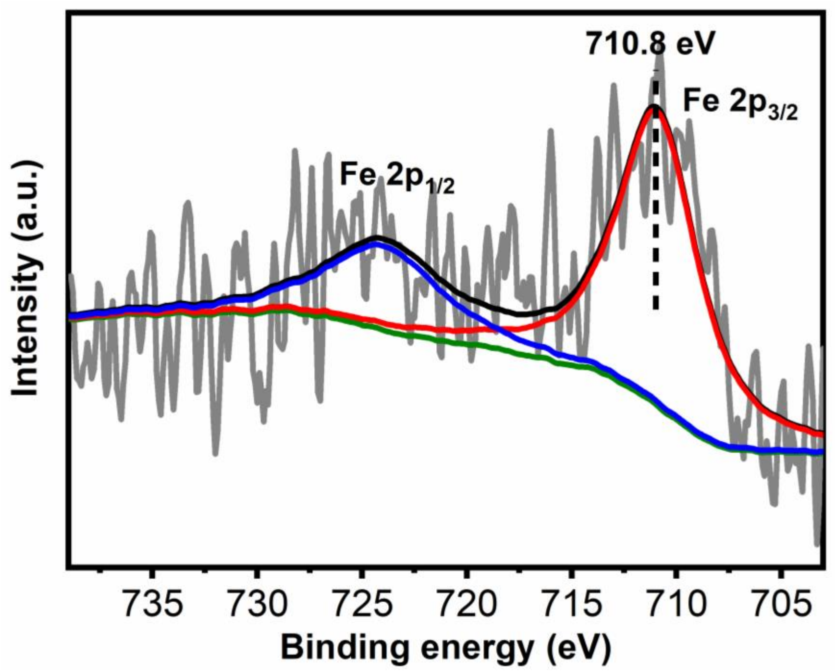

Supplementary Figure 27 | XPS spectrum. Fe 2p spectrum of Fe_{SA}-N-C after stability test.

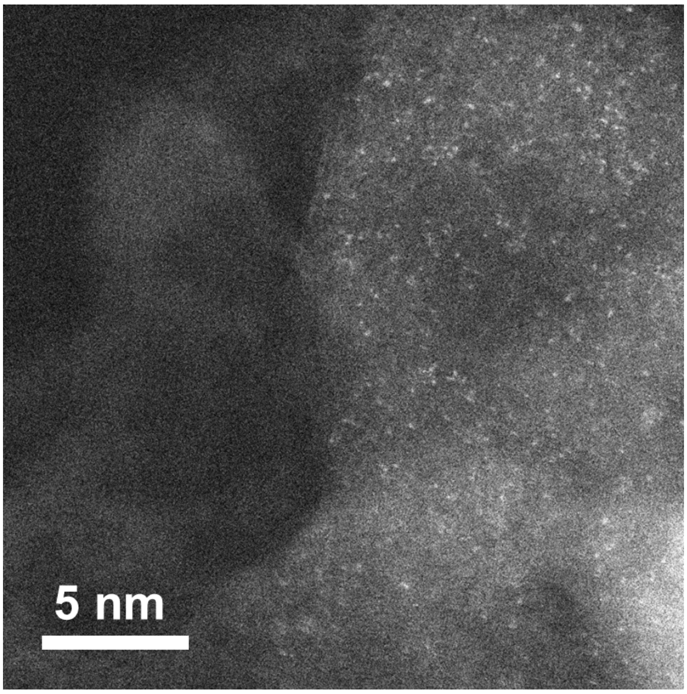

Supplementary Figure 28 | Aberration-corrected HAADF-STEM image of Fe_{SA}-N-C after stability test.

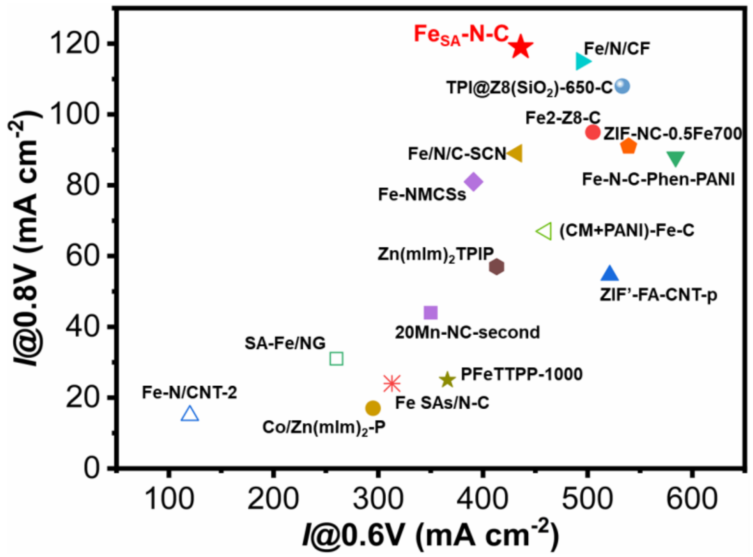

Supplementary Figure 29 | Comparison of acidic fuel cell performance between Fe$_{SA}$-N-C and other non-noble metal electrocatalysts under Department of Energy (DOE) reference conditions.

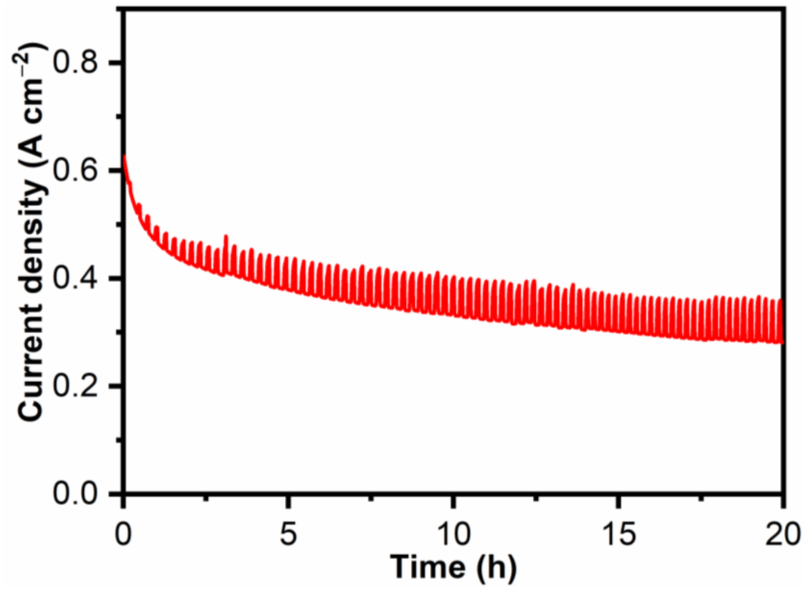

Supplementary Figure 30 | Fuel cell stability test of the Fe$_{SA}$-N-C-based membrane-electrode assembly (MEA). A typical decay phenomenon for highly active Fe$_{SA}$-N-C;[49] that is, a fast decay in the initial several hours followed by a stabilized current density ~ 0.3 A cm^{-2}.

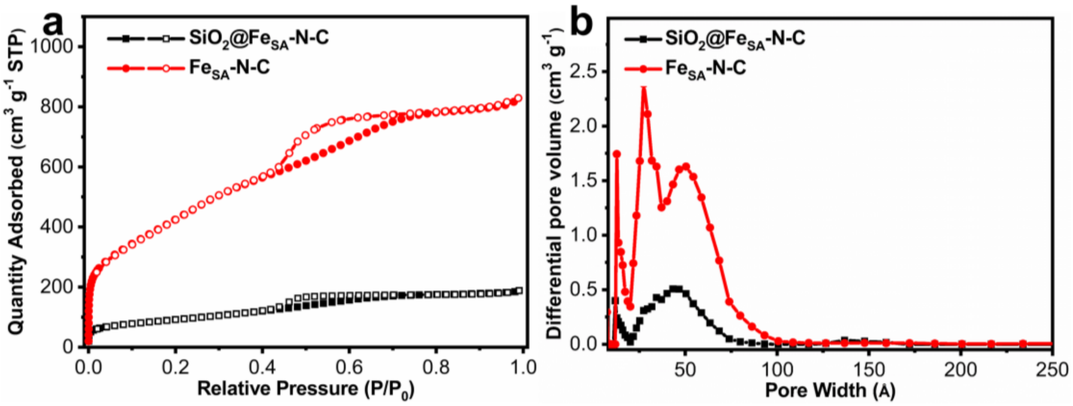

Supplementary Figure 31 | Comparison of N_2 sorption isotherm and pore-size distribution curves between SiO_2@Fe_{SA}-N-C and Fe_{SA}-N-C. (a) N_2 sorption isotherm at 77 K of SiO_2@Fe_{SA}-N-C and Fe_{SA}-N-C and (b) the corresponding pore size distribution calculated by the DFT model.

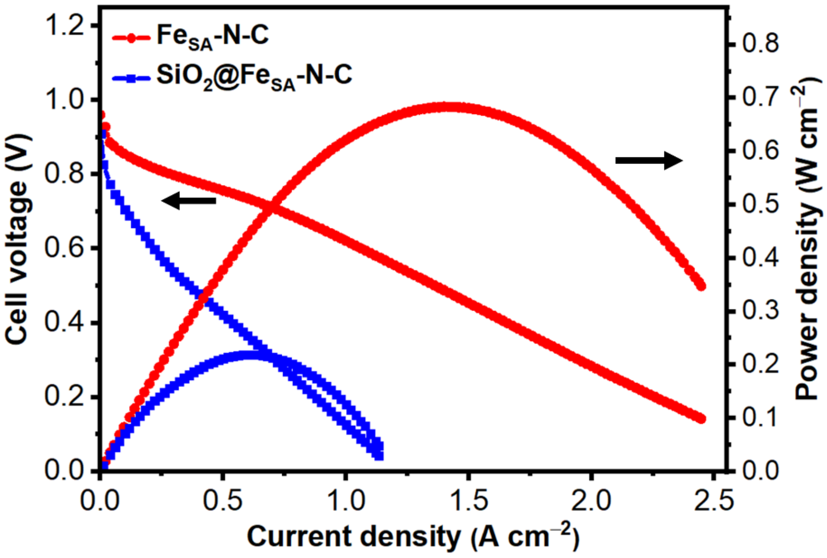

Supplementary Figure 32 | Fuel cell performance comparison of SiO_2@Fe_{SA}-N-C and Fe_{SA}-N-C. Polarization and power density curves of SiO_2@Fe_{SA}-N-C (Fe: 2.02 wt%) and Fe_{SA}-N-C (Fe: 3.46 wt%) cathode catalysts.

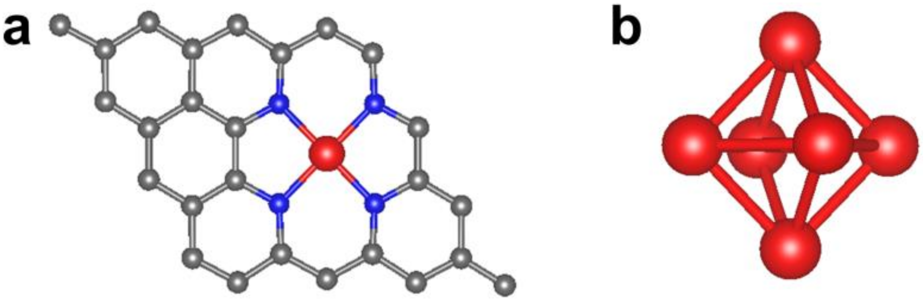

Supplementary Figure 33 | Computational models of (a) Fe_{SA}-N-C and (b) Fe_6 particle for ORR.

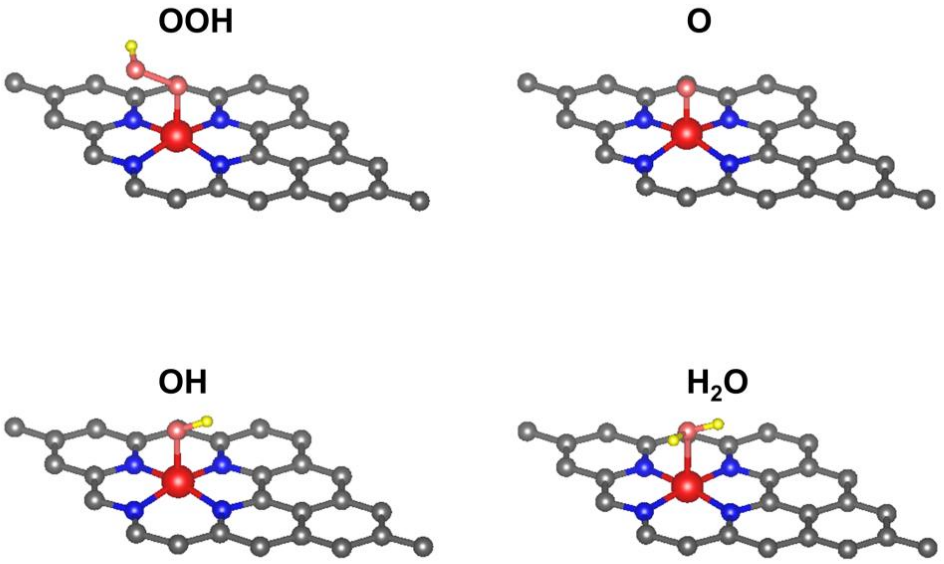

Supplementary Figure 34 | Configurations of adsorbates on Fe_{SA}-N-C.

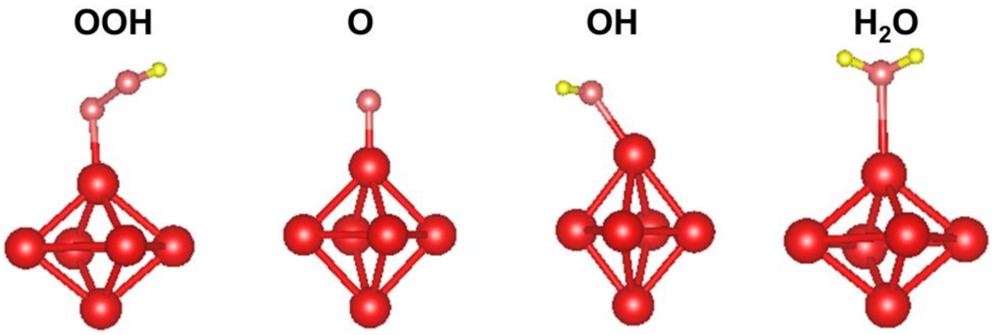

Supplementary Figure 35 | Configurations of adsorbates on Fe_6 particle.

Supplementary Table 1 | Comparison of PCN-222(Fe) synthesized by different monocarboxylic acid modulators.

	modulator	T_b (℃)[a]	pre-activation by HCl(aq)	mesopore size (nm)	Ref.
**PCN-222**	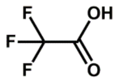 **CF_3COOH**	**72.4**	**without**	**3.2**	**This work**
	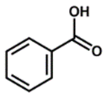 C_6H_5COOH	249	without	2.8	*Angew. Chem. Int. Ed.* 2012, 51, 10307.
			pre-activated by dilute acid	3.2	

[a] T_b: boiling temperature of modulator.

Supplementary Table 2 | Comparison of Fe loading between Fe_{SA}-N-C and other reported single-atom Fe loaded N-doped carbon materials.[a]

Sample	Fe content	Ref
**Fe_{SA}-N-C**	**3.46 wt %**	**This work**
**TPI@Z8(SiO_2)-650-C**	2.78 wt%	*Nat. Catal.* **2019**, 2, 259.
**Fe_{SA}-N-C**	1.09 wt%	*Nat. Commun.* **2019**, *10*, 341.
**Fe-N-C HNSs**	1.4 wt%	*Adv. Mater.* **2019**, *31*, 1806312.
**Fe-N/C-CNTs**	0.50 wt %	*ACS Catal.* **2019**, *9*, 336.
**Fe-NHGF**	0.2 wt%	*Nat. Catal.* **2018**, *1*, 63.
**Fe-SAs/NPS-HC**	1.54 wt%	*Nat. Commun.* **2018**, *9*, 5422.
**Fe SAs-N/C-20**	0.20 wt %	*J. Am. Chem. Soc.* **2018**, *140*, 11594.
**FeN_4/GN**	4.0 wt %	*Chem* **2018**, *4*, 1902.
**Fe-N_4 SAs/NPC**	1.96 wt %	*Angew. Chem. Int. Ed.* **2018**, *57*, 8614.
**Fe_{SA}-N-C**	1.76 wt%	*Angew. Chem. Int. Ed.* **2018**, *57*, 8525.
**Fe-ISA/SNC**	0.95 wt%	*Adv. Mater.* **2018**, *30*, 1800588.
**NDC-900**	0.46 wt%	*Adv. Energy Mater.* **2018**, *8*, 1701771.
**FeCl1N4/CNS**	1.5 wt%	*Energy Environ. Sci.* **2018**, *11*, 2348.
**Fe-N-C-950**	0.32 wt %	*ACS Catal.* **2018**, *8*, 2824.
**Fe-N-C**	0.91 wt%	*Small* **2018**, *14*, 1704282
**ISA Fe/CN**	2.16 wt%.	*Angew. Chem. Int. Ed.* **2017**, *56*, 6937.
**NDC-900**	0.18 wt%	*Adv. Energy Mater.* **2017**, *8*, 1701771.
**Fe-N-C-600**	1.8 wt%	*J. Am. Chem. Soc.* **2017**, *139*, 10790.
**SA-Fe/CN.**	0.9 wt%	*J. Am. Chem. Soc.* **2017**, *139*, 10976.
**Fe©N-C-12**	0.37 wt%	*ACS Catal.* **2017**, *7*, 7638.
**C-AFC©ZIF-8**	0.64 wt%	*Nano Energy* **2017**, *38*, 281.
**FePhenMOF-ArNH_3**	0.5 wt%	*Energy Environ. Sci.* **2016**, *9*, 2418.

[a] All data recorded here were based on inductively coupled optical emission spectrometer (ICP) data.

Supplementary Table 3 | The contents of Fe and Zr in Fe_{SA}-N-C quantified by different methods.

	Fe	Zr	Fe/Zr molar ratio
ICP	3.46 wt%[b]	0.09 wt%	62:1
XPS[a]	4.09 wt% (0.92 at%[c])	0.14 wt% (0.02 at%)	46:1
EDS	4.25 wt%	0.16 wt%	43:1

[a] The atomic percentage of elements were converted to mass percentage to make better comparison; [b] wt%: mass percentage; [c] at%: molar/atomic percentage.

This table clearly presents the high loading of Fe, extremely low content of Zr, and the large Fe/Zr molar ratio in Fe_{SA}-N-C. This table, together with the EXAFS spectrum of Fe_{SA}-N-C, can prove that the high-density bright dots in HADDF-STEM images of Fe_{SA}-N-C is Fe atoms rather than Zr atoms.

Supplementary Table 4 | Parameters of the best Fe K-edge EXAFS Fitting results for Fe_{SA}-N-C.

Catalysts	shell	CN	R (Å)	σ^2 (10^{-3}Å^2)	ΔE (eV)	R factor	*K*-Range (Å^{-1})	R-Range (Å)
**Fe_{SA}-N-C**	**Fe-N**	**3.5**	**1.95**	**9.79**	**-8.935**	**0.0072**	**2.5 - 10.8**	**1.06 -2**

Amplitude reduction factor S_0^2 is determined to be 0.81 through fitting the FT-EXAFS of standard Fe foil which is measured simultaneously during the experience. CN, coordination number; R, distance between absorber and backscatter atoms; σ^2, Debye-Waller factor to describe the variance in due to disorder (both lattice and thermal); ΔE, threshold Energy Correction; R factor (%) indicates the goodness of the fit.

Supplementary Table 5 | Comparison of ORR performance for various catalysts in 0.1 M KOH.[a]

Catalysts	Mass activity@0.9 V
Fe_{SA}-N-C	21.36 mA mg^{-1}
Fe_{NP}-N-C	2.88 mA mg^{-1}
N-C	0.41 mA mg^{-1}
Pt/C	0.14 A mg_{Pt}^{-1}

[a] The mass activity for Fe_{SA}-N-C, Fe_{NP}-N-C and N-C are calculated by the total mass of the catalyst. The mass activity for Pt/C is calculated based on the metal loading of Pt/C.

Supplementary Table 6 | Comparison of ORR performance between Fe_{SA}-N-C and other non-noble metal electrocatalysts in 0.1 M KOH solution.

Catalyst	$E_{1/2}$ (V *vs.* RHE)	J_k@0.85 V (mA cm^{-2})	J_L (mA cm^{-2})	year	Ref.
Fe_{SA}-N-C	0.90	37.19	5.75	2019	This work
Fe–NC SAC	0.90	39.2	5.6	2019	11
Fe-N-C HNSs	0.87	9.2	5.9	2019	12
Fe SAs-N-C	0.91	36.25*	5.8*	2019	13
Zn/CoN-C	0.86	2.47*	6.1	2019	13
Fe2-Z8-C	0.87	9.65*	6*	2018	15
SA-Fe/NG	0.88	3.36	5.6	2018	16
Fe_{SA}–N–C	0.89	6	23.27	2018	17
FeSAs/PTF-600	0.87	7.63	5.51	2018	18
Cu-N-C	0.87	11.84	5.7	2018	19
Mn/C-NO	0.86	5.9	5.7	2018	20
Fe-ISAs/CN	0.9	37.83*	6*	2017	21
CNT/PC	0.88	22.9*	4.59*	2016	22
FePhen@MOF-ArNH_3	0.86	7.93*	5.45*	2015	23
Fe_3C/NG-800	0.86	6.66*	5.8*	2015	24
FeCo/C-800	0.85	2.99*	5.2*	2015	25
Fe-N/C-800	0.81*	1.97*	4.8*	2015	26
(Fe,Mn)-N-C	0.87	16.1*	5.5*	2015	27
Co-N-C	0.87	17.33*	5.2*	2015	28
Co-Zn-ZIF/GO-800	0.84	2.99*	5.2	2015	29
Fe-N/C-800	0.8	2.17*	6.09	2014	30
S/N-Fe_{27}	0.87*	12.3*	5.7*	2014	31

$E_{1/2}$: half-wave potential; J_k@0.85 V: kinetic current density at 0.85 V; J_L: limitting current density.

Supplementary Table 7 | Comparison of ORR performance for various catalysts in 0.1 M $HClO_4$.[a]

	Mass activity@0.9 V
Fe_{SA}-N-C	1.12 mA mg_{cat}^{-1}
Fe_{NP}-N-C	0.06 mA mg_{cat}^{-1}
N-C	0.17 mA mg_{cat}^{-1}
Pt/C	0.1 A mg_{Pt}^{-1}

[a] The mass activity for Fe_{SA}-N-C, Fe_{NP}-N-C and N-C are calculated by the total mass of the catalyst.

Supplementary Table 8 | Comparison of ORR performance between Fe_{SA}-N-C and other non-noble metal electrocatalysts in 0.1 M $HClO_4$ solution.[a]

Catalyst	$E_{1/2}$ (V *vs.* RHE)	J_k@0.80 V (mA cm^{-2})	J_L (mA cm^{-2})	year	Ref.
**Fe_{SA}-N-C**	**0.80**	**6.14**	**5.60**	**2019**	**This work**
**Fe SAs-N-C**	0.79	4.78*	5.7*	2019	13
**Zn/CoN-C**	0.79	1.43*	6.3	2019	14
**Fe-N/CNT-2**	0.77	2.65*	5.6*	2019	32
**Cr/N/C-950**	0.76	0.55*	5.4*	2019	33
**SA-Fe/NG**	0.80	2.1	5.2	2018	16
**Fe-N-C-950**	0.78	2.7*	5.7*	2018	34
**Fe_{SA}–N–C**	0.78	3.32	5.7	2018	17
**FeSAs/PTF-600**	0.75*	1.54	5.42	2018	18
**Fe-ISAs/CN**	0.78*	2.61*	5.8*	2017	21
**CNT/PC**	0.79	4.5*	6*	2016	22
**Fe-NMCS**	0.72*	0.8*	5.6*	2016	35
**FePhen@MOF-$ArNH_3$**	0.77	2.63*	5.61*	2015	23
**CAPNI-Fe-NaCl**	0.73	0.7	5.1	2015	36
**FeCo/C-800**	0.76	1.37*	5.5*	2015	25
**Co-N-C**	0.76	1.2*	6.0	2015	28
**Co-Zn-ZIF/GO-800**	0.71*	0.21*	4.2	2015	29
**Fe-N/C-800**	0.66	0.31	6.06	2014	30
**$Zn(eIm)_2$TPIP**	0.78	2.35*	5*	2014	37
**PFeTTPP-1000**	0.76	1.02*	5*	2013	38
**C−N−Co**	0.79	3*	4.5	2013	39

$E_{1/2}$: half-wave potential; J_k@0.80 V: kinetic current density at 0.80V; J_L: limitting current density.

[a] To avoid performance loss caused by bisulfate adsorption on Pt/C, we employed 0.1 M $HClO_4$ rather than 0.5 M H_2SO_4 as the electrolyte for ORR test in acid. To make the data comparison more convincingly, we collected the data tested in 0.1 M $HClO_4$ specifically for the comparison of ORR in acidic condition.

Supplementary Table 9 | Comparison of acidic PEMFC performance between Fe_{SA}-N-C and other non-noble metal electrocatalysts under DOE reference conditions.[a,b]

Catalyst	I@0.6 V (mA cm^{-2})	P@0.6 V (mW cm^{-2})	I@0.8 V (mA cm^{-2})	P@0.8 V (mW cm^{-2})	Ref.
**Fe_{SA}-N-C**	**436**	**261.6**	**119**	**95.2**	**This work**
**Fe2-Z8-C**	505	303	95	76	15
**ZIF'-FA-CNT-p**	521	312.6	54.6	43.6	40
**Fe-N-C-Phen-PANI**	584	350.4	88	70.4	41
**Fe-NMCSs**	391	234.6	81	64.8	35
**Fe/N/C-SCN**	432	259.2	89	71.2	42
**Fe/N/CF**	494	296.4	115	92	43
**$Zn(mIm)_2$ TPIP**	413	247.8	57	45.6	37
**PFeTTPP-1000** [a]	366	219.6	25	20	38
**ZIF-NC-0.5Fe700**[a]	539	323.4	91	72.8	44
**TPI@Z8(SiO_2)-650-C**	533	319.8	108	86.4	45
**(CM+PANI)-Fe-C**	460	276	67	53.6	46
**Fe SAs/N-C**	313	187.8	24	19.2	13
**Fe-N/CNT-2**	120	72	15	12	32
**SA-Fe/NG**	260	156	31	24.8	16
**20Mn-NC-second**	350	210	44	35.2	47
**Co/$Zn(mIm)_2$-P**	295	177	17	13.6	48

I@0.6 V: current density at 0.6 V; P@0.6 V: power density at 0.6 V; I@0.8 V: current density at 0.8 V; P@0.8 V: power density at 0.8 V.

[a] Considering the US DOE standard test for platinum-group-metals-free catalyst, the PEMFC current density are all corrected to the value at 1 bar according to the equation below:[40]

$$I^* = I\ \left(\frac{P_{O_2}^*}{P_{O_2}}\right)^{0.79}\left(\frac{P_{H_2}^*}{P_{H_2}}\right)^{\alpha_c/2}$$

Where $P_{O_2}^*$ and $P_{H_2}^*$ are the reference pressures, α_c is the cathodic transfer coefficient and is assumed to be 1.

[b] Quantitatively, the current density at 0.8 V was used as an indicator to estimate the intrinsic ORR

activities of the nonprecious-metal catalysts. Another important parameter is the current density at 0.6 V, which is a reasonable voltage for practical operation. Thus, the data at 0.8 V and 0.6 V are summarized.

Supplementary Table 10 | Reaction energetics for the 4-elctron transfer processes during ORR.

Elementary reactions	ΔG*	
	Fe-N_4	Fe_6
$O_2 + H^+ + e^- + * = OOH*$	0.54	-0.24
$OOH* + H^+ + e^- = O* + H_2O$	-1.35	-1.59
$O* + H^+ + e^- = OH*$	0.56	0.58
$OH* + H^+ + e^- = H_2O$	0.25	1.25

*ΔG: free energy change at T=298 K, pH=1 and U= 0 V.

Supplementary Table 11 | Parameters of the DFT calculations for Fe_{SA}-N-C. Frequencies (cm^{-1}), zero point energies (ZPE), and entropy contribution (T*S) for different intermediates on Fe_{SA}-N-C (T = 298K).

	Frequencies (cm^{-1})	ZPE (eV)	T*S (eV)
O_2*	1273.01, 336.46, 167.16, 136.72, 60.88, 54.60	0.15	0.00
OOH*	3635.03, 1270.04, 786.32, 447.87, 303.90, 231.78, 150.96, 91.34, 78.52	0.43	0.00
O*	764.52, 164.20, 160.02	0.07	0.00
H_2O*	3810.50, 3696.56, 1580.14, 375.08, 315.11, 149.38, 88.96, 65.60, 46.43	0.64	0.00
OH*	3689.63, 839.65, 496.40, 139.38, 123.88, 100.23	0.35	0.00
H_2O (g)	1621, 3739, 3856	0.57	0.60
H_2 (g)	4408	0.27	0.42
O_2 (g)	1558	0.10	0.62

Supplementary Table 12 | Parameters of the DFT calculations for Fe_6. Frequencies (cm^{-1}), zero point energies (ZPE), and entropy contribution (T*S) for different intermediates on Fe_6 (T = 298K).

	**Frequencies (cm^{-1})**	**ZPE (eV)**	**T*S (eV)**
O_2*	1282.15, 463.73, 269.13, 210.62, 98.12, 84.18	0.12	0.00
OOH*	3616.10, 1268.15, 830.55, 555.19, 270.04, 262.87, 171.74, 97.67, 87.11	0.42	0.00
O*	808.21, 173.77, 171.71	0.07	0.00
H_2O*	3742.30, 3629.65, 1519.25, 491.13, 422.19, 333.63, 173.59, 83.53, 57.76	0.63	0.00
OH*	3645.96, 885.50, 580.88, 283.85, 150.57, 148.99	0.32	0.00
H_2O (g)	1621, 3739, 3856	0.57	0.60
H_2 (g)	4408	0.27	0.42
O_2 (g)	1558	0.10	0.62

Supplementary References

[1] Ravel, B. & Newville, M. ATHENA, ARTEMIS, HEPHAESTUS: data analysis for X-ray absorption spectroscopy using IFEFFIT. *J. Synchrotron Radiation.* **12**, 537-541 (2005).

[2] Zitolo, A. *et al.* Identification of catalytic sites for oxygen reduction in iron- and nitrogen-doped graphene materials. *Nat. Mater.* **14**, 937-942 (2015).

[3] Feng, D. *et al.* Zirconium-metalloporphyrin PCN-222: mesoporous metal-organic frameworks with ultrahigh stability as biomimetic catalysts. *Angew. Chem. Int. Ed.* **51**, 10307-10310 (2012).

[4] Asano, N., Uemura, S., Kinugawa, T., Akasaka, H. & Mizutani, T. Synthesis of biladienone and bilatrienone by coupled oxidation of tetraarylporphyrins. *J. Org. Chem.* **72**, 5320-5326 (2007).

[5] Kresse, G. & Joubert, D. From ultrasoft pseudopotentials to the projector augmented-wave method. *Phys. Rev. B* **59**, 1758-1775 (1999).

[6] Perdew, J. P., Burke, K. & Ernzerhof, M. Generalized gradient approximation made simple. *Phys. Rev. Lett.* **77**, 3865-3868 (1996).

[7] Kresse, G. & Furthmüller, J. Efficiency of ab-initio total energy calculations for metals and semiconductors using a plane-wave basis set. *Comput. Mater. Sci.* **6**, 15-50 (1996).

[8] Kresse, G. & Furthmüller, J. Efficient iterative schemes for ab initio total-energy calculations using a plane-wave basis set. *Phys. Rev. B* **54**, 11169-11186 (1996).

[9] Nørskov, J. K. *et al.* Origin of the overpotential for oxygen reduction at a fuel-cell cathode. *The J. Phys. Chem. B* **108**, 17886-17892 (2004).

[10] Haynes, William M. CRC handbook of chemistry and physics. *CRC press* (2014).

[11] Zhao, L. *et al.* Cascade anchoring strategy for general mass production of high-loading single-atomic metal-nitrogen catalysts. *Nat. Commun*. **10**, 1278 (2019).

[12] Chen, Y. F. *et al.* Atomic Fe dispersed on N-doped carbon hollow nanospheres for high-efficiency electrocatalytic oxygen reduction. *Adv. Mater.* **31**, 1806312 (2019).

[13] Yang, Z. K. *et al.* Boosting oxygen reduction catalysis with Fe–N_4 sites decorated porous carbons toward fuel cells. *ACS Catal.* **9**, 3, 2158-2163 (2019).

[14] Lu, Z. Y. *et al.* An isolated zinc–cobalt atomic pair for highly active and durable oxygen reduction. *Angew. Chem. Int. Ed.* **58**, 2648-2652 (2019).

[15] Liu, Q., Liu, X., Zheng, L. R. & Shui, J. L. The solid-phase synthesis of an Fe-N-C electrocatalyst for high-power proton-exchange membrane fuel cells. *Angew. Chem. Int. Ed.* **57**, 1204-1208 (2018)

[16] Liu, Y. *et al.* Unveiling the high-activity origin of single-atom iron catalysts for oxygen reduction reaction. *Proc. Natl. Acad. Sci.* **115**, 6626-6631 (2018).

[17] Jiao, L. *et al.* From metal–organic frameworks to single-atom Fe implanted N-doped porous carbons: efficient oxygen reduction in both alkaline and acidic media. *Angew. Chem. Int. Ed.* **57**, 8525-8529 (2018).

[18] Yi, J. D. *et al.* Atomically dispersed iron–nitrogen active sites within porphyrinic triazine-based frameworks for oxygen reduction reaction in both alkaline and acidic media. *ACS Energy Lett*. **3**, 883-889 (2018).

[19] Li, F. *et al.* Boosting oxygen reduction catalysis with abundant copper single atom active sites. *Energy Environ. Sci.* **11**, 2263-2269 (2018).

[20] Yang, Y. *et al.* O-, N-atoms-coordinated Mn cofactors within a graphene framework as bioinspired oxygen reduction reaction electrocatalysts. *Adv. Mater.* **30**, 1801732 (2018).

[21] Chen, Y. J. *et al.* Isolated single iron atoms anchored on N-doped porous carbon as an efficient electrocatalyst for the oxygen reduction reaction. *Angew. Chem. Int. Ed.* **56**, 6937-6941 (2017).

[22] Sa, Y. J. *et al.* A general approach to preferential formation of active Fe–Nx sites in Fe–N/C electrocatalysts for efficient oxygen reduction reaction. *J. Am. Chem. Soc.* **138**, 15046−15056 (2016).

[23] Strickland, K. *et al.* Highly active oxygen reduction non-platinum group metal electrocatalyst without direct metal–nitrogen coordination. *Nat. commun.* **6**, 7343 (2015).

[24] Xiao, M. L., Zhu, J. B., Feng, L. G., Liu, C. P. & Xing, W. Meso/macroporous nitrogen-doped carbon architectures with iron carbide encapsulated in graphitic layers as an efficient and robust catalyst for the oxygen reduction reaction in both acidic and alkaline solutions. *Adv. Mater.* **27**, 2521 -2527 (2015).

[25] Lin, Q. P. *et al.* Heterometal-embedded organic conjugate frameworks from alternating monomeric iron and cobalt metalloporphyrins and their application in design of porous carbon catalysts. *Adv. Mater.* **27**, 3431-3436 (2015).

[26] Niu, W. H. *et al.* Mesoporous N-doped carbons prepared with thermally removable nanoparticle templates: An efficient electrocatalyst for oxygen reduction reaction *J. Am. Chem. Soc.* **137**, 5555-5562 (2015).

[27] Sahraie, N. R. *et al.* Quantifying the density and utilization of active sites in non-precious metal oxygen electroreduction catalysts. *Nat. Commun.* **6**, 8618 (2015).

[28] You, B., Jiang, N., Sheng, M. L., Drisdell, W. S., Yano, J. & Sun, Y. J. Bimetal–organic framework self-adjusted synthesis of support-free nonprecious electrocatalysts for efficient oxygen reduction. ACS Catal. **5**, 7068-7076 (2015).

[29] Wei, J. *et al.* Nitrogen-doped nanoporous carbon/graphene nano-sandwiches: synthesis and application for efficient oxygen reduction. *Adv. Funct. Mater.* **25**, 5768-5777 (2015).

[30] Lin, L., Zhu, Q. & Xu, A. W. Noble-metal-free Fe–N/C catalyst for highly efficient oxygen reduction reaction under both alkaline and acidic conditions. *J. Am. Chem. Soc.* **136**, 11027-11033 (2014).

[31] Sahraie, N. R., Paraknowitsch, J. P., Göbel, C., Thomas, A. & Strasser, P. Noble-metal-free electrocatalysts with enhanced ORR performance by task-specific functionalization of carbon using ionic liquid precursor systems. *J. Am. Chem. Soc.* **136**, 14486-14497 (2014).

[32] Xia, D. S. *et al.* Direct growth of carbon nanotubes doped with single atomic Fe–N_4 active sites and neighboring graphitic nitrogen for efficient and stable oxygen reduction electrocatalysis. *Adv. Funct. Mater.* **29**, 1906174 (2019).

[33] Luo, E. *et al.* Single-atom Cr-N_4 sites designed for durable oxygen reduction catalysis in acid media. *Angew. Chem. Int. Ed.* **58**, 12469-12475 (2019).

[34] Xiao, M. L. *et al.* Microporous framework induced synthesis of single-atom dispersed Fe-N-C acidic ORR catalyst and its in situ reduced Fe-N_4 active site identification revealed by X-ray absorption spectroscopy. *ACS Catal.* **8**, 2824-2832 (2018).

[35] Meng, F. L., Wang, Z. L., Zhong, H. X., Wang, J., Yan, J. M. & Zhang, X. B. Reactive multifunctional template-induced preparation of Fe-N-doped mesoporous carbon microspheres towards highly efficient electrocatalysts for oxygen reduction. *Adv. Mater.* **28**, 7948–7955 (2016).

[36] Ding, W. *et al*. Shape fixing via salt recrystallization: a morphology-controlled approach to convert nanostructured polymer to carbon nanomaterial as a highly active catalyst for oxygen reduction reaction. *J. Am. Chem. Soc.* **137**, 5414-5420 (2015).

[37] Zhao, D. *et al.* Highly efficient non-precious metal electrocatalysts prepared from one-pot synthesized zeolitic imidazolate frameworks. *Adv. Mater.* **26**, 1093–1097 (2014).

[38] Yuan, S. et al. A highly active and support-free oxygen reduction catalyst prepared from ultrahigh-surface-area porous polyporphyrin. *Angew. Chem. Int. Ed.* **52**, 8349–8353 (2013).

[39] Liang, H. W., Wei, W., Wu, Z. S., Feng, X. & Müllen, K. Mesoporous metal–nitrogen-doped carbon electrocatalysts for highly efficient oxygen reduction reaction, *J. Am. Chem. Soc.* **135**, 16002-16005 (2013).

[40] Zhang, C. *et al.* Networking pyrolyzed zeolitic imidazolate frameworks by carbon nanotubes improves conductivity and enhances oxygen-reduction performance in polymer-electrolyte-membrane fuel cells. *Adv. Mater.* **29**, 1604556 (2017).

[41] Fu, X. G. *et al.* In situ polymer graphenization ingrained with nanoporosity in a nitrogenous electrocatalyst boosting the performance of polymer-electrolyte-membrane fuel cells. *Adv. Mater.* **29**, 1604456 (2017).

[42] Wang, Y. C. *et al.* S-doping of an Fe/N/C ORR catalyst for polymer electrolyte membrane fuel cells with high power density. *Angew. Chem. Int. Ed.* **54**, 9907–9910 (2015).

[43] Shui, J. L., Chen, C., Grabstanowicz, L., Zhao, D. & Liu, D. J. Highly efficient nonprecious metal catalyst prepared with metal–organic framework in a continuous carbon nanofibrous network**.** *Proc. Natl. Acad. Sci.* **112**, 10629–10634 (2015).

[44] Li, J. Z. *et al.* Thermally driven structure and performance evolution of atomically dispersed FeN_4 sites for oxygen reduction. *Angew. Chem. Int. Ed.* **58**, 2-12 (2019).

[45] Wan, X. *et al.* Fe–N–C electrocatalyst with dense active sites and efficient mass transport for high-performance proton exchange membrane fuel cells. *Nat. Catal.* **2**, 259–268 (2019).

[46] Chung, H. T *et al.* Direct atomic-level insight into the active sites of a high-performance PGM-free ORR catalyst. *Science.* **357**, 479-484 (2017).

[47] Li, J. Z. *et al.* Atomically dispersed manganese catalysts for oxygen reduction in proton-exchange membrane fuel cells. *Nat. Catal.* **1**, 935-945 (2018).

[48] Chong, L. *et al.* Investigation of oxygen reduction activity of catalysts derived from Co and Co/Zn methyl-imidazolate frameworks in proton exchange membrane fuel cells. *ChemElectroChem*. **3**, 1541 (2016).

[49] Chenitz, R. *et al.* A specific demetalation of Fe-N_4 catalytic sites in the micropores of NC_Ar+NH_3 is at the origin of the initial activity loss of the highly active Fe/N/C catalyst used for the reduction of oxygen in PEM fuel cells. *Energy Environ. Sci.* **11**, 365-382 (2018).
